# Positive Darwinian selection in the singularly large taste receptor gene family of an ‘ancient’ fish, *Latimeria chalumnae*

**DOI:** 10.1186/1471-2164-15-650

**Published:** 2014-08-05

**Authors:** Adnan S Syed, Sigrun I Korsching

**Affiliations:** Institute of Genetics, University of Cologne, 50674 Cologne, Germany

**Keywords:** Coelacanth, Bitter taste, Pheromone, Phylogeny, Sarcopterygian, Evolution

## Abstract

**Background:**

Chemical senses are one of the foremost means by which organisms make sense of their environment, among them the olfactory and gustatory sense of vertebrates and arthropods. Both senses use large repertoires of receptors to achieve perception of complex chemosensory stimuli. High evolutionary dynamics of some olfactory and gustatory receptor gene families result in considerable variance of chemosensory perception between species. Interestingly, both *ora/v1r* genes and the closely related *t2r* genes constitute small and rather conserved families in teleost fish, but show rapid evolution and large species differences in tetrapods. To understand this transition, chemosensory gene repertoires of earlier diverging members of the tetrapod lineage, i.e. lobe-finned fish such as *Latimeria* would be of high interest.

**Results:**

We report here the complete T2R repertoire of L*atimeria chalumnae*, using thorough data mining and extensive phylogenetic analysis. Eighty *t2r* genes were identified, by far the largest family reported for any species so far. The genomic neighborhood of *t2r* genes is enriched in repeat elements, which may have facilitated the extensive gene duplication events resulting in such a large family. Examination of non-synonymous *vs*. synonymous substitution rates (dN/dS) suggests pronounced positive Darwinian selection in *Latimeria* T2Rs, conceivably ensuring efficient neo-functionalization of newly born *t2r* genes. Notably, both traits, positive selection and enrichment of repeat elements in the genomic neighborhood, are absent in the twenty *v1r* genes of *Latimeria*. Sequence divergence in *Latimeria* T2Rs and V1Rs is high, reminescent of the corresponding teleost families. Some conserved sequence motifs of Latimeria T2Rs and V1Rs are shared with the respective teleost but not tetrapod genes, consistent with a potential role of such motifs in detection of aquatic chemosensory stimuli.

**Conclusions:**

The singularly large T2R repertoire of Latimeria may have been generated by facilitating local gene duplication *via* increased density of repeat elements, and efficient neofunctionalization *via* positive Darwinian selection.

The high evolutionary dynamics of tetrapod *t2r* gene families precedes the emergence of tetrapods, i.e. the water-to-land transition, and thus constitutes a basal feature of the lobe-finned lineage of vertebrates.

**Electronic supplementary material:**

The online version of this article (doi:10.1186/1471-2164-15-650) contains supplementary material, which is available to authorized users.

## Background

Chemosensation is an ancient sense, its origins going all the way back to unicellular organisms. In vertebrates and arthropods, two specialized senses have evolved. The olfactory sense serves a host of essential functions, among them search for food or prey, predator evasion, mate choice and reproduction, kin recognition and signalling of social status, whereas the gustatory sense is tasked with vital decisions about safety and desirability of food sources. Neuronal representation and the logic of coding sensory input are very different for vertebrate taste and smell [1–4]. Olfactory sensory neurons form one (teleost fish), two (lungfish, amphibians) or several (mammals) extended sensory epithelia, and directly project to the (rostral) brain, whereas small clusters of taste cells (taste buds) are found distributed across several nonsensory epithelia (oral cavity, gills, skin for teleost fish), and their innervating neurons connect to (caudal) brain stem neurons. Moreover, different receptor families serve olfaction and taste [1–3]. Olfactory receptor genes are typically expressed in monogenic fashion, whereas co-expression of receptors shapes the response characteristics of taste cells [1–3]. All these differences notwithstanding, closely related families do segregate between these two senses, gustatory T2Rs *vs*. olfactory V1Rs, and T1Rs *vs*. V2Rs, respectively.

Basic features of olfactory and gustatory representation appear to be conserved across vertebrates [3–7]. However, the high evolutionary dynamics of olfactory and gustatory receptor gene families allows for considerable variance in neuronal representation of chemosensory signals between species [8]. In particular, the relative importance of different chemosensory receptor gene families appears to have changed drastically between tetrapods and teleosts [9–12]. Teleost fish species possess only very small *t2r* gene families, whereas a much larger variability has been observed in tetrapods, with up to 50 genes in an amphibian species [11]. Even more strikingly, the V1R-related *ora* gene repertoires of teleosts consist of the same six genes, with an occasional gene loss [10], whereas mammalian *v1r* gene repertoires are highly species-specific [13].

It has been proposed that chemosensory receptor family sizes adapt to the particular ecological environment of each species. Mammalian T2Rs and at least one fish T2R signal bitter taste [2, 14], and bitter substances often occur as chemical defense mechanism of plants. Accordingly it has been suggested that the size of the T2R repertoire is larger in herbivorous than in carnivorous species [15]. Mammalian V1Rs are assumed to detect volatile pheromones [16], which could be related to the larger size and higher species specificity of mammalian V1R families. In contrast, the homologous ORA family of fishes is expected to detect hydrophilic substances, which may serve a different biological function. To examine such hypotheses it would be useful to establish the corresponding receptor repertoires of aquatic species from the tetrapod lineage.

Teleosts belong to the ray-finned lineage of vertebrates, whereas mammals and other tetrapods belong to the lobe-finned lineage, which also includes fish like coelacanths of the genus *Latimeria* and lungfish as very early diverging representatives [17]. One might expect the *v1r* and *t2r* gene repertoires of lobe-finned fishes to resemble those of ray-finned fishes more that those of land-living tetrapods. Alternatively, the higher evolutionary dynamics observed for tetrapods could be a common feature of the lobe-finned lineage of vertebrates. Recently, the genome of the coelacanth *Latimeria chalumnae* has been published [18], but initial gene searches have resulted in highly contradictory results, showing either a teleost-like small T2R repertoire of only 5 genes [15] or a large amphibian-like repertoire of 58 genes [19]. The V1R family size has alternatively been given as 15 or 20 genes [19, 20]. To clarify these discrepancies, we performed a thorough bioinformatic analysis of the *Latimeria chalumnae* genome to delineate and characterize the *t2r* and *v1r* gene repertoires in this species.

We report here that *Latimeria* possess an unequaled large *t2r* gene repertoire of eighty genes that exhibit strong evidence for positive Darwinian selection, and whose genomic neighborhood shows increased density of repeat elements. Both these features are absent in the closely related *Latimeria* V1Rs, which nevertheless show much less negative selective pressure than their teleost counterparts. Together, these findings indicate that high evolutionary dynamics of *t2r* and *v1r* gene families are not linked to the loss of aquatic life style in tetrapods, but appear to be an ancient evolutionary characteristic of the lobe-finned lineage.

## Results

**Figure 1:**
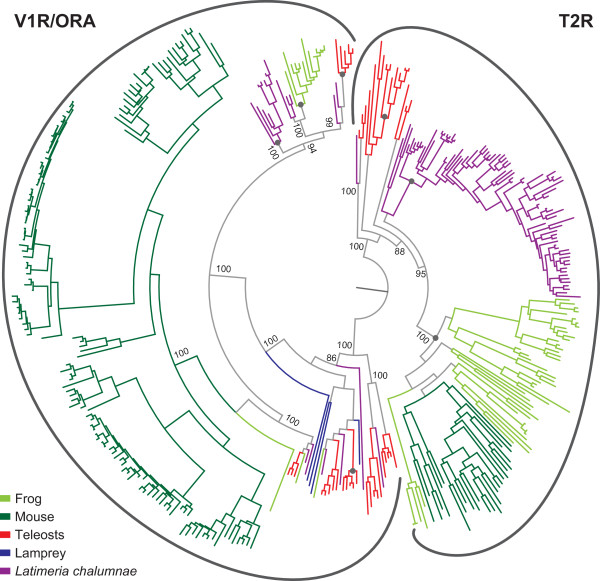
**Monophyletic origin of the T2R and the V1R/ORA receptor family.** The phylogenetic tree was generated using a maximum likelihood method (PhyML-aLRT) with SPR setting for tree optimization and chi square-based aLRT for branch support (given as percentage). Branches are color-coded for the respective species (*Latimeria chalumnae*, purple; mouse, dark green; *Xenopus tropicalis*, light green; lamprey, blue; 5 teleost fish species [zebrafish, stickleback, medaka, fugu, tetraodon], red). *t2r* of all species form a single subclade, as do all *v1r* genes. Grey filled circles indicate clades analysed for evidence of positive selection, see Figure 5. Gene sequences for *Xenopus tropicalis* and teleosts were taken from [11] for T2Rs and [10] for V1R/ORAs.

To delineate the Latimeria *t2r* and *v1r* gene repertoires we performed a recursive search of the preliminary draft of the *Latimeria chalumnae* genome [18] provided by the Broad Institute [21], using representative T2R and V1R/ORA protein sequences from mouse, frog (*Xenopus tropicalis*) and zebrafish as initial queries. No additional candidates were found searching an independently sequenced Latimeria genome [20]. Candidate genes were evaluated by phylogenetic analysis, using a maximum-likelihood method, PhyMl-aLRT [22]. Published T2R and V1R/ORA sequences from lamprey (*Petromyzon marinus*), five teleost fish species, frog, and mouse were used as reference. Since *t2r* genes constitute the closest neighbors of *v1r/ora* genes, each group served as stringent outgroup for the other one. In initial analyses additional outgroups were used to delineate the combined V1R + T2R group of genes from other rhodopsin-like GPCRs, with very similar results.We observe a clear-cut segregation with very high branch support between a monophyletic T2R and a monophyletic V1R/ORA group (Figure 1). This allows to unambiguously assign candidate genes to the respective family.

### An unprecedentedly large T2R repertoire results from extensive gene duplications of a single ancestral t2r gene

**Figure 2:**
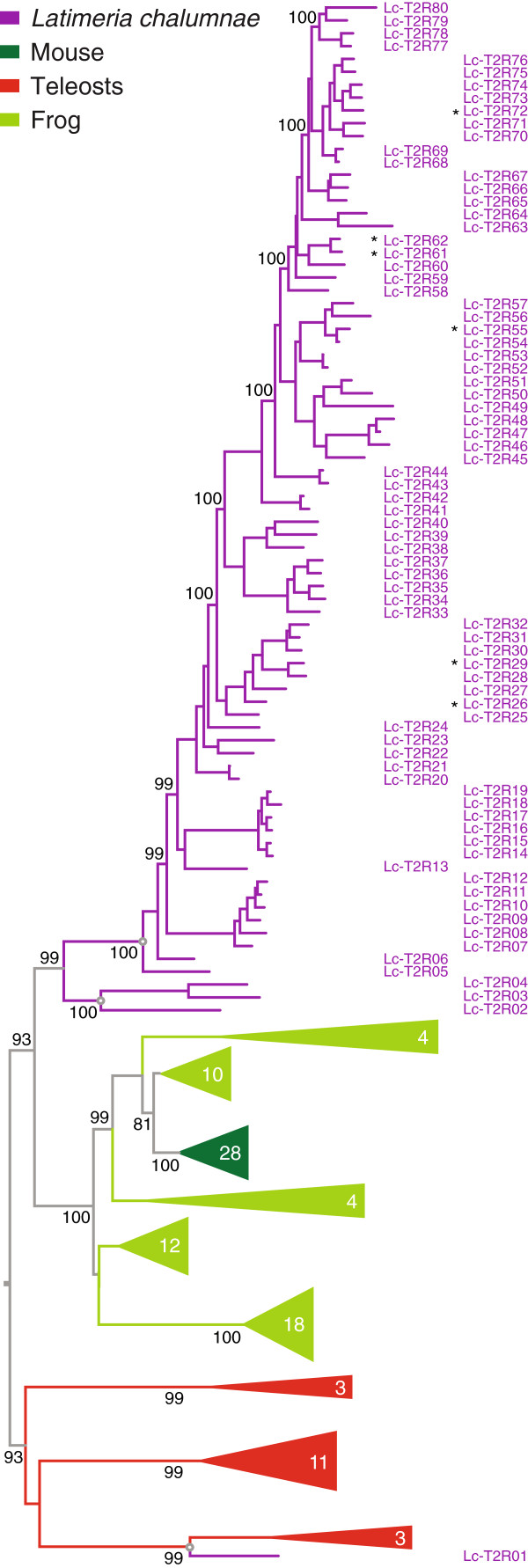
**Three ancestral genes and a single large expansion in the Latimeria T2R family.** Eighty T2R receptors of *Latimeria* were compared with T2R receptors of mouse, *Xenopus tropicalis*, and 5 teleost fishes (species and color code as given for Figure 1). The phylogenetic tree was generated as described for Figure 1; branch support is given as percentage. Asterisks, potential pseudogenes, see Methods for details. For accession numbers and genomic location of *Latimeria* genes see Additional file 1. Three ancestral genes are indicated by open circles at the respective nodes.

Eighty *t2r* genes were identified in the *Latimeria* genome (Figures 1 and 2, Additional file 1 and Additional file 2), by far the largest repertoire found in any species so far, nearly double the size of the largest previously reported repertoire, *Xenopus tropicalis* (49 genes, [11]). Seventyfive of these *Latimeria t2r* genes have been missed in a recent multi-species study [15], possibly because validation criteria used there have eliminated many *bona fide t2r* genes. Twentytwo of *Latimeria t2r* genes have been missed in a recent multi-family study [19] that seems to have investigated only previously predicted genes, which in our experience [10, 12, 23, 24] does not result in complete coverage of a chemosensory family. Our approach is comprehensive and does not rely on any prior annotation whatsoever, as our inclusion criterion is based solely on phylogenetic position of candidate genes, see Methods. Six of the 80 genes we report contain up to 2 stop codons and may either represent pseudogenes or databank inaccuracies. 74 genes have been validated as full length, and all 80 genes contain the expected motifs (see also Methods, and below).

The vast majority of *Latimeria t2r* genes (Lc_T2R05 to Lc_T2R80) appear to result from a single ancestral gene *via* extensive gene duplications (Figure 2). Another ancestral gene only went through 2 duplication events, resulting in Lc_T2R02 to Lc_T2R04, and no gene duplication was observed for Lc_T2R01, the third ancestral *Latimeria t2r* gene. We would like to point out that Lc_T2R01 is also the only *Latimeria t2r* gene with any ortholog in other species. Three teleost *t2r* genes, stickleback T2R3, puffer T2R1, and fugu T2R1 are direct orthologs of Lc_T2R01 (100% branch support, Figure 2). As such, Lc_T2R01 represents the first available evidence for a common origin of individual teleost and tetrapod *t2r* genes. In total, *Latimeria chalumnae* appears to possess three ancestral genes (Figure 2), two of which were subject to species-specific gene expansions. The extent of one of these gene duplications is unparalleled in any species investigated so far, but nevertheless places the Latimeria T2R family in the vicinity of tetrapod T2R repertoires, and far away from teleost T2R repertoires, which only comprise 3–6 genes [11].

### The Latimeria V1R family possesses close orthologs/paralogs of all six teleost ora genes, but also exhibits several gene expansions characteristic of tetrapod V1R repertoires

**Figure 3:**
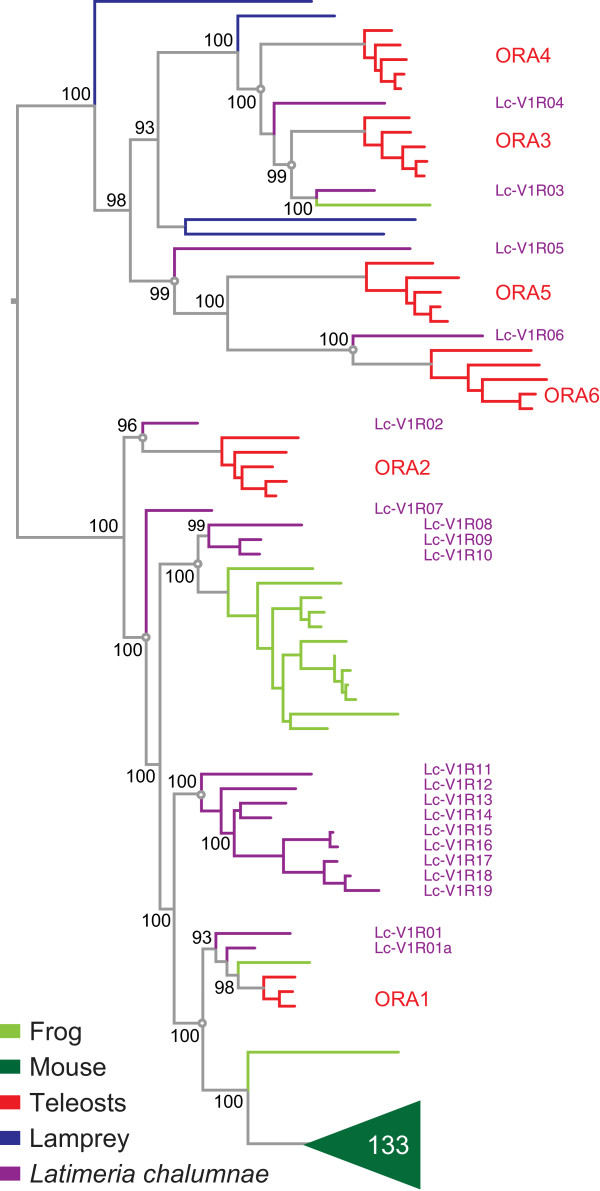
**Teleost/tetrapod hybrid characteristics of the Latimeria V1R family.** Twenty V1R receptors of *Latimeria* were compared with V1R and V1R-related ORA receptors of mouse, *Xenopus tropicalis*, lamprey and 5 teleost fishes (species and color code as given for Figure 1). The phylogenetic tree was generated as described for Figure 1. Branch support is given as percentage. For accession numbers and genomic location of *Latimeria* and lamprey genes see Additional file 1. Ancestral genes are indicated by open circles at the respective nodes.

Twenty *ora*-related *v1r* genes were identified in the *Latimeria* genome (Figures 1 and 3, Additional files 1 and 2), consistent with results of a recent phylogenetic study using data from an independent genome sequencing approach [20]. We expect this number to be very close to final, even though the genome assembly is still in draft stage [18], since the genome has been sequenced with high coverage (61 fold, [18]), and our gene identification approach is not sensitive to assembly quality. Phylogenetic analysis shows nine ancestral genes (Figure 3), six of which are shared with teleost fish (Lc_V1R01-06), and indeed three of these genes (Lc_V1R02, 03, 06) constitute direct one-to-one orthologs of the corresponding teleost *ora* genes, e.g. Lc_V1R02 is ortholog to ORA2 and so forth. The remaining 3 ancestral nodes are all located within the ORA1/ORA2 subclade, and exhibit varying degrees of gene expansion, similar to observations for later-derived species in the lobe-finned lineage such as frogs and mammals, *cf*. [25]. A small group of three *Latimeria v1r* genes (Lc_V1R08 to Lc_V1R10) emerges as sister clade to the main gene expansion in frog, whereas a larger group of 9 *Latimeria v1r* genes (Lc_V1R11 to Lc_V1R19) is more closely related to the (single) mammalian subclade of *v1r* genes (Figure 3, cf. [10]). These two gene expansions appear to have occurred independently within the *Latimeria* lineage, i.e. after divergence from the most recent common ancestor (MRCA) shared with tetrapods. Taken together, the *Latimeria* V1R repertoire shows the divergence characteristic of teleost ORA families and the gene expansion characteristic for tetrapod V1R families.

### Motif analysis validates the phylogenetic assignment of Latimeria v1r and t2r genes and shows considerable species-specific conservation

T2R sequence identities can exceed 90% in pairwise comparisons, and the same holds true for pairwise comparisons of V1R sequences (*cf*. Additional file 1), consistent with an origin of such genes by recent gene duplications. However, overall both gene families are highly heterogenous, with frequent identity values between 40 to 50% and minimal identities down to 23% for T2R, and 19% for V1R sequences (Additional file 1). It therefore appeared instructive to analyse the evolution of conserved sequence motifs of T2R and V1R families in the tetrapod lineage, and to compare it to the teleost lineage. To the best of our knowledge such motif analysis comparing V1R and T2R families has not been performed in any species so far.

**Figure 4:**
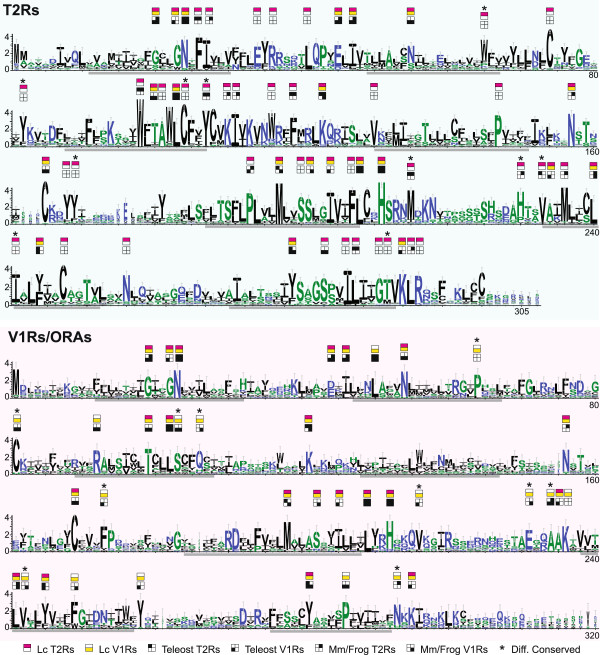
**Motif analysis for Latimeria T2R and V1R/ORA receptors confirms results of the phylogenetic analysis.** Sequence logos for 80 *Latimeria* T2Rs and 20 *Latimeria* V1Rs are shown. Letter height indicates the relative frequency with which a particular amino acid appears at that position. Amino acid conservation above 45% is indicated as follows: *Latimeria* T2Rs, magenta rectangle; *Latimeria* V1Rs, yellow rectangle; other T2Rs, upper black squares (teleost, left; mouse and frog, right); other V1Rs, lower black squares (teleost, left; mouse and frog, right). White squares and rectangles, no conservation found; asterisk, the same position is differently conserved between *Latimeria* T2Rs and V1Rs; grey bars, transmembrane domains.

Thus we constructed separate multiple sequence alignments for tetrapod T2Rs (mouse and frog), tetrapod V1Rs (mouse and frog), teleost T2Rs and teleost V1R-related ORAs, visualized them as sequence logos [26], and compared them with those of *Latimeria* V1Rs and T2Rs (Figure 4, Additional file 1). Over 70 highly and moderately conserved amino acids were identified, organized in motifs of 1 to 3 amino acids, among them some motifs conserved in several GPCR families, and many motifs shared between *Latimeria* V1Rs and T2Rs, as expected from the close phylogenetic relationship of these two families (Figure 4). We identified 14 amino acid positions that are conserved in tetrapod and/or teleost *t2r*, but not in *v1r* genes. All but one show the same specificity in *Latimeria*. Furthermore, many amino acids are solely conserved in *Latimeria* T2Rs (22 amino acids) and two amino acids are only conserved in *Latimeria* V1R. In one case, the loss of the generally conserved cysteine in EC1 of *Latimeria* T2Rs is compensated by a cysteine in n-8 position, conserved only in *Latimeria* T2Rs. Either cysteine may form a disulfide bridge with a broadly conserved cysteine in EC2. Finally, ten positions are conserved differentially in T2Rs *vs*. V1Rs (Figure 4, Additional file 1). All these observations support the phylogeny-based assignment of *Latimeria t2r* and *v1r* genes (Figure 4, Additional file 1).

Amino acids that are differentially conserved between T2R and V1R receptors, e.g. Y_T2R_/C_V1R_ in EC1, and C..Y_T2R_/S..Q_V1R_ in TM3, may be expected to be relevant for the functional differences between T2R and V1R receptors, and would be plausible candidates for a functional analysis by site-directed mutagenesis in future studies. In several cases residues conserved in *Latimeria* T2Rs and/or V1Rs are only conserved in either the teleost or the tetrapod lineages, e.g. a *Latimeria* T2R-specific KI motif in the IC2 region that is conserved in tetrapod T2Rs, but not in teleost T2Rs (Figure 4). Examples for motifs conserved in teleost T2Rs, but not in tetrapod T2Rs include a central Y in TM6 and in TM7 (Figure 4). Such pattern of conservation is consistent with *Latimeria* genes keeping features of the posited ancestral genes, that were differentially retained in later-deriving members of the lobe-finned lineage (tetrapods) and the ray-finned lineage (teleosts). It remains to be seen, whether residues shared with teleost, but not with tetrapod V1Rs and T2Rs, might be specifically relevant for aquatic chemosensation.

Overall, however, a high degree of divergence is visible within *Latimeria* T2Rs and within *Latimeria* V1Rs. Such high divergence might be generated by positive Darwinian selection, which has been shown to occur in several chemosensory receptor gene families [12, 27–29]. We have therefore examined nucleotide substitution ratios to obtain an estimate for positive selection in *Latimeria t2r* and *v1r* gene families.

### Pronounced positive selection in the T2R family suggested by dN/dS analysis

We compared the rate of **n**onsynonymous (dN) to **s**ynonymous (silent) nucleotide substitutions (*dN/d*S) separately for all codons, to obtain an estimate for the evolutionary constraints acting on the *v1r* and *t2r* gene families of *Latimeria*. A value below 1 for dN/dS indicates negative selective pressure, i.e. purifying selection, whereas values larger than 1 suggest positive selection, i.e. selection for diversity [30]. dN/dS = 1 equals neutral selection. To avoid distortion of the dN/dS ratio by beginning saturation of synonymous substitutions [31] the dS values should not exceed a certain value, differently given as 2 or 3 [32]. We therefore verified that this condition was met for all *Latimeria* genes (dS < 0.5) and all genes from species we examined for comparative purposes (frog T2R and V1R frog, dS < 0.6; teleost fish T2R, dS < 0.5; teleost ORA2 and ORA4, 2.5 and 1.5, respectively). In order to obtain a stringent measure of positive selection we employ two different algorithms, single likelihood ancestor counting (SLAC) and fixed effects likelihood (FEL) to estimate dN/dS, and only report sites, for which both methods give the same prediction with a probability better than threshold, p < 0.1 (*cf*. [33].

**Figure 5:**
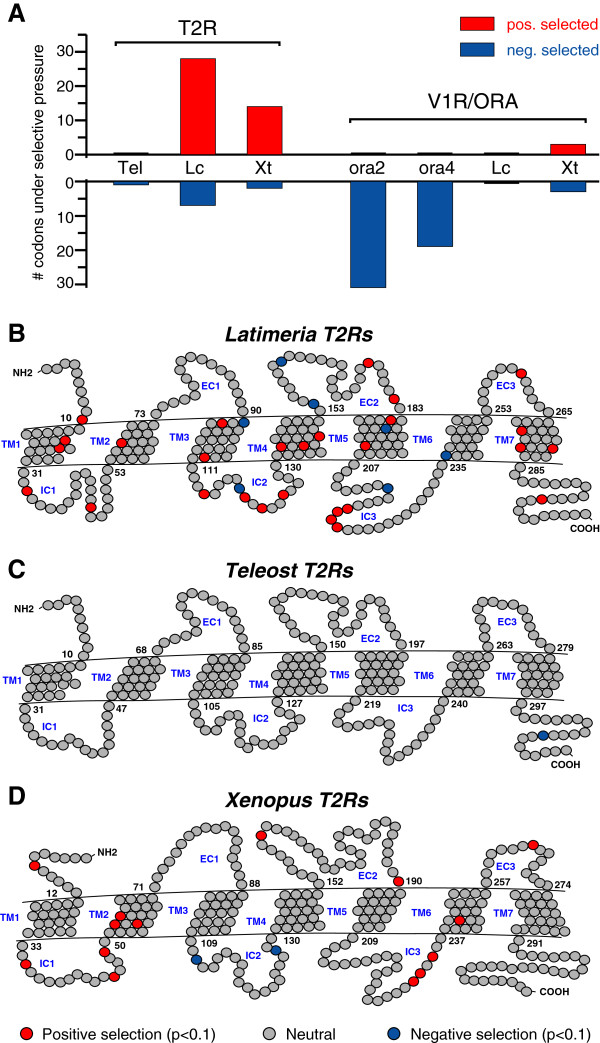
**Evidence for extended positive selection in Latimeria t2r genes.** Selective pressure for individual codons is shown as consensus of single likelihood ancestor counting (SLAC) and fixed effects likelihood (FEL) algorithm as implemented on the datamonkey server [62]. **A)** Number of positively and negatively selected sites (p < 0.1) for six clades depicted in Figure 1; tel, 7 teleost T2Rs; Lc, 76 Lc_T2Rs; Xt, 49 Xt_T2Rs; 5 ORA2; 5 ORA4, 9 Lc_V1Rs; 12 Xt_V1Rs. **B-D)** Results for T2R clades are presented as snake plot (negative selection in blue, *p* < 0.1, neutral selection in gray, positive selection in red, *p* < 0.1). **B)** Latimeria T2R sequences. Twentyeight codons show evidence for positive selection both with SLAC and FEL algorithms. **C)** Teleost T2R sequences. No evidence for positive selection. **D)** Frog T2R sequences. Fourteen codons show evidence for positive selection with both algorithms.

We observe an impressive number of 28 positively selected sites in the *Latimeria t2r gene*s, and a much smaller number of negatively selected sites (Figure 5A, Additional file 1). This is twice the number of positively selected sites in frog *t2r* genes (Figure 5A), and suggests a high evolutionary dynamic in Latimeria *t2r* genes, which is unexpected, since *Latimeria* genes generally are evolving slowly [34]. Many of the positively selected sites even show p values below 0.01 (Additional file 1). Positively selected sites are situated in extra- and intracellular compartments as well as most transmembrane regions (Figure 5B). A small cluster of 4 contiguous positively selected sites occurs in the third intracellular loop, and another accumulation of four sites is observed in the preceding intracellular loop. A high variability in these loops could either diversify the interaction with signalling molecules or indirectly influence the positions of the transmembrane regions, which are believed to constitute the binding pocket for tastants [35]. Nearly half of the positively selected sites (13 of 28) are within the transmembrane domains (Figure 5B), not significantly different from frog T2Rs (4 of 14 sites, p > 0.2, chi square test), and at least some of these sites could exert a direct influence on ligand binding.

In contrast, teleost *t2r* genes do not exhibit a single positively selected site (Figure 5A), suggesting that selection for diversity may be a characteristic feature of taste receptor evolution in the lobe-finned, but not the ray-finned lineage.

### The V1R family exhibits neither pronounced positive nor negative selection

Teleost *ora* genes show very pronounced negative selection consistent with previous reports [10], whereas *Latimeria* and frog *v1r* genes exhibit no or nearly no negatively selected sites (Figure 5, Additional file 1). However, overall *v1r/ora* genes appear to be under higher evolutionary constraints than *t2r* genes, since we observe only rare positively selected sites in frog and none in Latimeria *v1r* genes (Figure 5). For *Latimeria v1r* genes neither negatively nor positively selected sites were found using both prediction methods, although one of the methods suggests the presence of some negatively selected sites. Thus it remains unresolved, whether *Latimeria v1r* genes are truly under neutral selection, or merely under weak purifying selection, undetected by the stringent search criteria applied.

### Latimeria t2r and v1r genes are intronless

**Figure 6:**
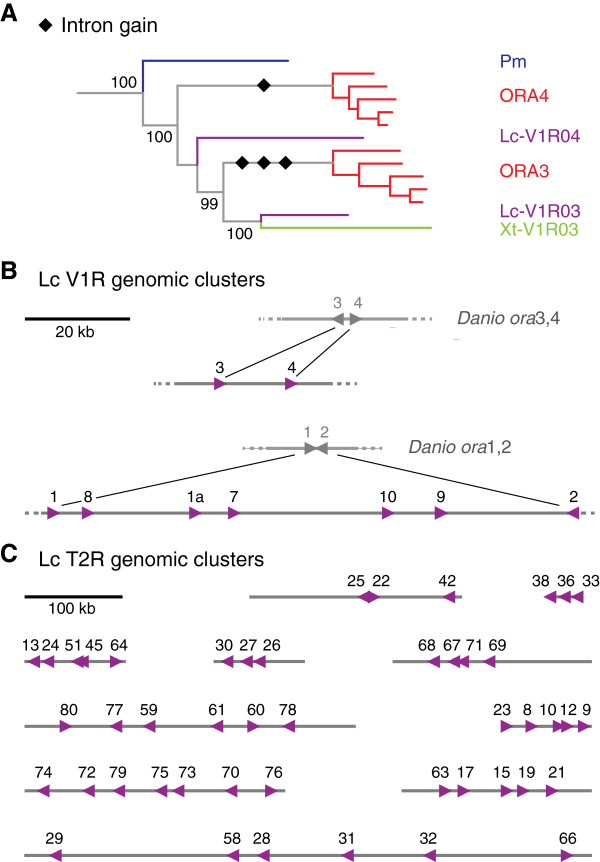
**Genomic structure and location of Latimeria v1r and t2r genes.** Panel **A**, phylogenetic origin of introns in the V1R/ora family. The subtree is taken from Figure 3; numbers indicate % branch support; black diamonds, intron gains. Panel **B**, comparison of the *Latimeria* V1R1/2 and V1R3/4 clades with the ORA1/2 and ORA3/4 gene pairs of teleosts. Numbers correspond to gene names; magenta triangles, *Latimeria* v1r genes; grey triangles, zebrafish genes; triangle pointing left, + strand; triangle pointing right, - strand; all gene distances drawn to scale. Panel **C**, *Latimeria t2r* gene clusters, full length of contigs is shown; numbers correspond to gene names; magenta triangles, *Latimeria* t2r genes; triangle pointing left, + strand; triangle pointing right, - strand; all gene distances drawn to scale. In comparison with an independent assembly [20], two contigs containing three and six genes (T2R25, 22, 42 and T2R29, 58, 28, 31, 32, 66, respectively), merge into a larger cluster of nine genes.

Mammalian T2Rs and V1Rs are monoexonic, while some teleost V1R-related ORAs are known to harbor 1 to 3 conserved introns [10]. We therefore evaluated all Latimeria T2R and V1R genomic sequences individually to obtain reliable exon/intron predictions. We find no evidence for introns in either gene family, including V1R03 and V1R04, orthologous respectively paralogous to intron-containing teleost *ora3* and *ora4* genes. Since the lamprey gene basal to both *v1r03*/*ora3* and *v1r04/ora4* is also intronless [36], we conclude that the intronless state is the ancestral feature, and that the intron gains resulting in polyexonic *ora3* and *ora4* genes have happened late in the vertebrate evolution, within the ray-finned lineage (Figure 6A).

### Intergenic distances between Latimeria t2r genes are larger than between Latimeria v1r genes

Despite the generally small size of T2R-containing contigs, three quarters of *t2r* genes are found with neighboring *t2r* genes. Also, over two thirds of *v1r* genes are found with neighboring *v1r* genes, allowing calculation of intergenic distances (Figure 6B,C). It is noteworthy that *t2r* genes, with their larger evolutionary dynamics (see above), exhibit also larger intergenic distances, 31 kb median value compared to 16 kb for *v1r* genes (*cf*. Additional file 1). For two teleost *ora* gene pairs (*ora1/2* and *ora3/4*) we compared the genomic arrangement of their four *Latimeria* orthologs/paralogs (Figure 6B). The teleost *ora*3/4 gene pair is locked in tail-to-tail orientation at few kb distance, *cf.* [10]. While the corresponding *Latimeria* genes *v1r03* and *v1r04* are also neighbors, they are severalfold further apart and have head-to-tail orientation. The Latimeria *v1r01* and *v1r02* genes, on the other hand, share the head-to-head orientation of their teleost counterparts *ora1* and *ora2*, but lie much farther apart, with about 100 kb distance between *v1r01* and *v1r02*. Five *v1r01*-related genes are located in the intervening sequence, all sharing the orientation of *v1r01*, suggesting that several gene duplications of the ancestral *v1r01* gene resulted in breaking the ancestral close association of *v1r01* and *v1r02* (Figure 6B).

### High density of repeat elements involved in gene duplication is observed close to t2r genes

**Figure 7:**
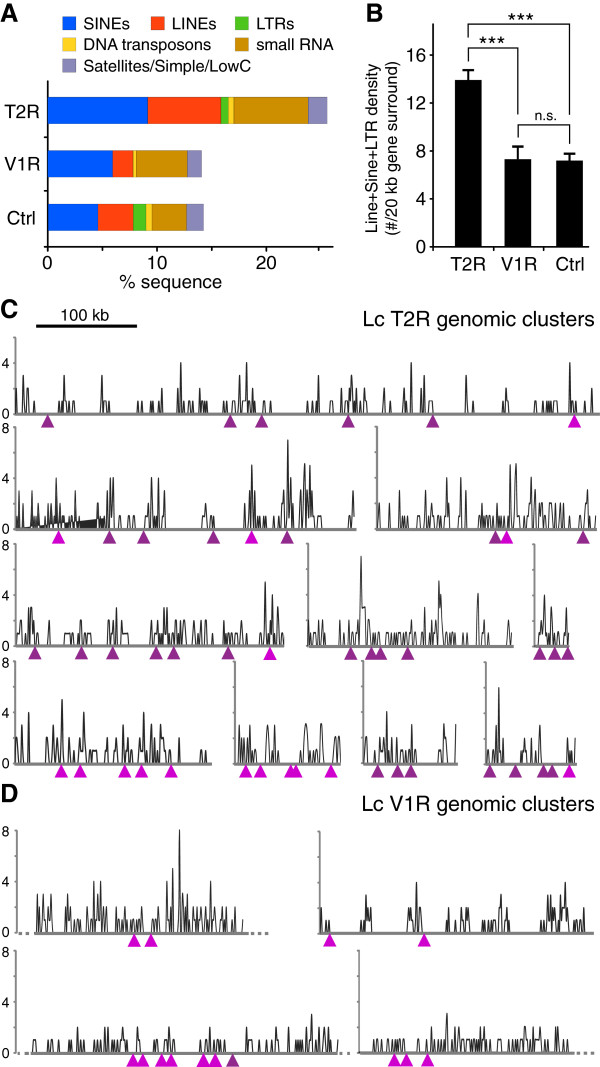
**Repeat elements are enriched in T2R, but not in V1R genomic neighborhood.** Transposable elements (repeat elements) were identified using RepeatMasker in ten *t2r* and four *v1r* gene clusters with up to 100 kb flanking regions and ten reference genomic regions (12.2 Mb total). **A)** Genomic sequence containing class I transposons (SINEs, LINEs, LTRs) and class II transposons (DNA transposons), small RNA, and Satellites/simple/low complexity repeats is determined as percent of total sequence in T2R and V1R clusters (each including 10 kb flanking regions) and the reference regions. Color code as indicated. **B)** Average density of class I transposons in 20 kb genomic segments centering on 47 *t2r* (T2R) and 14 *v1r* genes (V1R) and in 11.3 Mb reference region (Ctrl). Significance was evaluated by students t test (two-sided; ***, p < 0.001; n.s., not significant). **C, D)** The frequency of class I transposable elements is given as number of elements per kb sequence. The position of the genes is indicated by triangles; dark magenta, ++ (5′- > 3′); light magenta,+- (3′- > 5′). **C)** Ten contigs with *t2r* gene clusters are shown, from left to right and top to bottom, JH128085, JH128916, JH129648, JH129303, JH129750, JH132239, JH129824, JH130976, JH130917, JH130784. **D)** Four contigs with *v1r* gene clusters are shown, from left to right and top to bottom, JH126562, JH129249, JH126576, JH1276167.

Repeat elements may facilitate gene duplication by increasing the probability of illegitimate cross-over during meiosis. In particular LINE, SINE, and LTR elements (class I transposable elements, retrotransposons) have been shown to correlate with gene duplications and inversions [20, 37–39]. An increased density of such elements close to *t2r* genes conceivably could provide a mechanism for the genesis of the record-sized T2R family. We have therefore analysed the distribution of repeat elements in the neighborhood of T2R clusters. Since drastically different average values for the contribution of repeat elements to the genome have been reported for Latimeria [18, 20], we have generated a reference value ourselves, using ten randomly selected scaffolds. We find that on average T2R cluster regions (≥3 *t2r* genes) contain 4.6% more SINE sequence and 3.5% more LINE sequence than the reference regions (Figure 7A). LTR elements constitute a comparatively small proportion of all repeat elements, consistent with other reports [40], and show little difference between T2R cluster regions and reference regions (Figure 7A).

The strongest association of class I transposable elements with gene duplication is found within 5–10 kb distance from the respective genes [41]. In fact, there is evidence for duplication of such 5–10 kb regions for another class of chemosensory receptor genes [42]. We have therefore determined the density of LINE, SINE, and LTR elements in 20 kb sequence segments centered on each *t2r* gene that belongs to an identifiable cluster (47 genes) (Figure 7B). We report that the *t2r* surround regions exhibit a significantly higher density of LINE/SINE/LTR elements than the reference regions (13.9+/-0.8 *vs*. 7.2+/-0.6 elements/20 kb, respectively; mean+/-SEM, p < 0.001, two-sided t-test).

Finally we have determined for all *t2r* gene clusters the frequency of repeat elements in the entire contigs using small scale (1 kb) binning (Figure 7C). It is noticable that often pronounced peak frequencies occur in very close association to *t2r* genes, and on the other hand very few *t2r* genes are located in stretches of sequence devoid of repeat elements (Figure 7C). Taken together, analysis on three different length scales (gene cluster region, effective neighborhood range and 1 kb high resolution mapping) shows an enrichment in repeat elements in the genomic vicinity of *t2r* genes. These findings suggest that the high evolutionary dynamic of Latimeria *t2r* genes might be at least in part facilitated by an enrichment of class l transposons in the corresponding genomic regions.

### v1r gene clusters show no increase in surround density of repeat elements

The *Latimeria* V1R family exhibits only moderate gene expansion, compared to the T2R family. Therefore it appeared instructive to compare the density of repeat elements in the vicinity of *v1r* genes to that found in reference regions as well as T2R clusters. We find on average that regions with V1R clusters (≥2 *v1r* genes) show slightly reduced LINE and SINE levels (1.0% and 1.8% of sequence below reference levels, respectively, Figure 7A). LTR elements are nearly completely absent (Figure 7A).

In the detailed analysis of 20 kb surround regions of *v1r* cluster genes the average frequency of retrotransposons is not significantly different from that observed in reference regions (Figure 7B). In contrast, the difference to *t2r* surround regions is highly significant (p < 0.001, t-test). In the small scale analysis only 1 of the 14 *v1r* genes present in clusters is associated with a noticable peak frequency of repeat elements, although several such peaks do exist in the larger vicinity (Figure 7D).

In summary, on all levels of analysis the genomic neighborhood of *v1r* genes is similar to control regions, whereas neighborhood regions of *t2r* genes show significant increases above control levels. Indeed, the frequency of repeat elements in *t2r* gene surrounds is double as large as that observed in *v1r* gene surrounds. Thus, the increased repeat density surrounding *t2r* genes is not a general feature of chemosensory genes in *Latimeria*, but is correlated with the unusually large increase in the T2R family size during coelacanth evolution.

## Discussion

Coelacanths (*Latimeria*) are so-called living fossils, as they are one of the few extant fish in the lobe-finned lineage of vertebrates, from which all tetrapods emerged [17]. The fossil record shows remarkable morphological consistency since the early Devonian [43], consistent with a generally slow rate of molecular evolution in coelacanth genes [34]. Chemosensory receptor families are among the fastest evolving gene families [8], and thus we were interested in the evolutionary dynamics of such families in a coelacanth genome. In particular, two of these gene families, the closely related V1R/ORA and T2R families, are known to rapidly evolve in tetrapods [13], whereas the corresponding gene repertoires in teleost fish are small and highly conserved [10, 11]. It is worth pointing out that this tetrapod/teleost difference is gene family-specific and cannot be generalized, since in another chemosensory gene family opposing trends are observed [12]. The sparse information available for cartilaginous and jawless fish [44] suggests that the teleost V1R/ORA repertoires may correspond to the ancestral situation.

**Figure 8:**
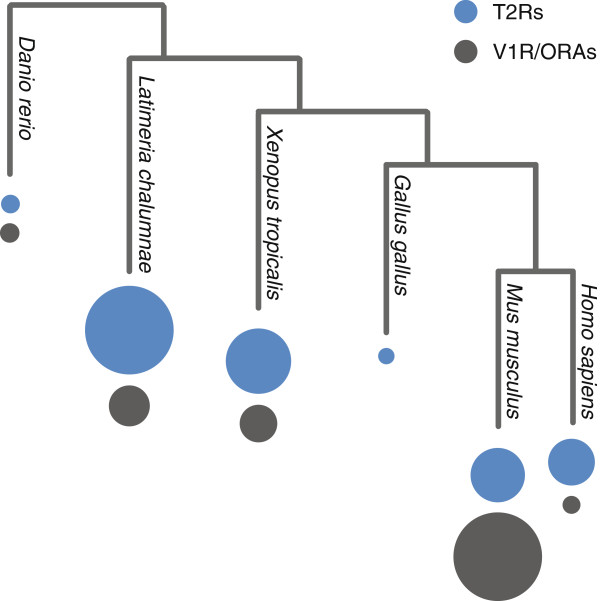
**Evolutionary dynamic of vertebrate t2r and v1r gene repertoires.** Species tree with the respective T2R (blue circle) and V1R/ORA (black circle) repertoires. Circle area is proportional to the size of the gene family. Values for *Latimeria* T2R and V1R families, this study; *t2r* gene family size for other species was taken from [11], teleost ORA and frog V1R family size from [10] and mouse V1R family size from [25].

We report here that *Latimeria chalumnae* possesses 80 *t2r gene*s, of which at least 74 are intact genes, which is by far the largest repertoire size reported for any species (Figure 8), and nearly double as much as that of the frog *Xenopus tropicalis*, the largest known repertoire so far [11]. The biological purpose for *Latimeria* of such a large T2R receptoire is unknown. T2R receptors are bitter taste receptors in mammals [2], possibly also in teleost fish [45] and are assumed to mediate avoidance of potentially toxic food sources. It has been suggested that herbivores would require larger T2R repertoires to guard them against plant chemical defense mechanisms [15]. This correlation is weakened by our results, since plants are absent in the habitat of *Latimeria*, the deep sea (*Latimeria* feeds on various fish and cephalopods [46]). However, it cannot be excluded that *Latimeria* T2Rs might have extra-gustatory functions, as has been shown for mammalian T2Rs [47].

The unparalleled size of the Latimeria T2R repertoire is unexpected, given the overall low mutation rate in *Latimeria* genes, *cf.* [34]. It is noteworthy that all but four of the *Latimeria t2r* genes are derived from a single ancestral gene, thus the diversification of the T2R repertoire seen here constitutes a recent development within this lineage. The increase in *Latimeria* T2R family size appears to have arisen by repeated local gene duplications, since the large majority of *t2r* genes are found in small clusters in several short contigs, which presumably will coalesce into larger cluster(s) as the genome assembly becomes more refined. In fact, a comparison with an independent sequencing effort [20] showed two of the clusters found here merging into a larger cluster. The significantly higher density of transposable elements in the immediate vicinity of *t2r* genes may provide a means to facilitate/enhance gene duplication in this gene family and could thus be part of the mechanism responsible for generating the large T2R family. Additionally, closely related neighboring *t2r* genes themselves might serve as recombination foci.

Genesis of a large gene repertoire requires not only gene duplications, but also an efficient path to neo-functionalization for these newly duplicated genes, which may involve positive selection. Indeed we found dN/dS values indicative of positive selection for a large number of sites localized in all three major compartments of the protein sequence (extracellular loops, transmembrane regions, intracellular loops), with small clusters in two intracellular loops. These sites might contribute either directly (sites in TMIII, TMV and TMVII, cf. [35]) or indirectly via overall conformational changes (sites in loops, other TMs) to diversification of *Latimeria* taste responses.

We wished to compare the extent of positive selection in *Latimeria t2r* genes to that observed in tetrapod and teleost chemosensory receptor families [12, 27, 48–50]. However, numerical comparison between results obtained by different algorithms is difficult, and so we also have examined dN/dS ratios for teleost and frog T2R repertoires here. We observe that Latimeria T2Rs by far show the most pronounced positive selection of all T2R families analysed, an unexpected result considering the generally low mutation rate in *Latimeria* genes, *cf.* [34]. We have also analysed V1R/ORA repertoires from *Latimeria*, teleost and frog, and did not find any evidence for positive selection in *Latimeria* V1Rs. Neither did *Latimeria* V1Rs exhibit the pronounced negative selection observed for the V1R-related *ora* genes of teleosts [10]. In other words, *Latimeria v1r* genes are drastically different from their teleost counterparts, and resemble more those of later diverging tetrapods (this manuscript, *cf.* [27, 48–50]. In all within-species comparisons, V1Rs exhibited either more negative or less positive selection than T2Rs. Taken together, *Latimeria*, an early-diverged and aquatic-living vertebrate species with generally slow evolution, shows evidence for (near) neutral evolution of its V1R and fast evolution of its T2R repertoire.

It has previously been hypothesized that the difference between (small) teleost and (large) tetrapod T2R and V1R repertoires might reflect an adaptation to the terrestrial lifestyle [11]. Furthermore these differences have been contrasted with the absence of such drastic changes in V2R (and T1R) repertoires, resulting in large changes of the ratio of *v1r* to *v2r* genes upon the acquisition of the terrestrial life style [25]. However, the results we report here for the *Latimeria* V1R family and in particular the *Latimeria* T2R family do not strengthen this hypothesis. *Latimeria* is a purely aquatic organism with a medium-sized V1R and very large T2R repertoire, whereas its V2R repertoire is comparable to that of teleost fishes (Korsching, unpublished observation). Consequently, the difference in size between teleost and tetrapod T2R repertoires is not related to the water-to-land transition. Instead, frequent gene birth events in particular in the T2R family appear to be a general feature of the lobe-finned lineage of vertebrates, and need to be understood in that context. Of course, this does not exclude an additional role, aquired much later, in facilitating the water-to-land transition.

On the other hand, for another parameter, sequence divergence, both the V1R and T2R receptor families of *Latimeria* examined here are more similar to those of teleosts than to those of later diverging members of their own lobe-finned lineage. While *Latimeria* possess sister clades to all mouse and frog *t2r* genes, they have additionally retained a ‘fish-like’ taste receptor, unlike mouse and frog. Furthermore, *Latimeria* exhibits direct orthologs or paralogs of all six teleost *ora* genes, in contrast to the amphibian *Xenopus*, who lost the majority, and mammals, who lost all direct orthologs, and kept paralogs of only two *ora* genes. Thus, the *Latimeria* T2R and V1R repertoires are more divergent than the corresponding repertoires of the later-derived tetrapods from the same (lobe-finned) lineage. The gradual loss of ancestral *v1r* genes in the lobe-finned lineage correlates with loss of aquatic life style (obligatory for *Latimeria*, facultative for *Xenopus*, and mostly absent in mammals) and conceivably these six highly conserved V1R/ORA receptors are specialized for detection of purely aquatic odor stimuli.

## Conclusions

Taken together we have shown hybrid features for the T2R and V1R receptor repertoire of a coelacanth, *Latimeria chalumnae.* Despite its basal position in the lobe-finned lineage, *t2r* genes of this species shows many species-specific gene duplications - conceivably facilitated by a high density of transposable elements - as well as evidence of positive Darwinian selection characteristic for later-diverged members of this lineage such as amphibians and mammals. At the same time, *Latimeria* retains most of the divergence characteristic of teleost chemosensory receptor repertoires, which to an increasing degree is lost in more modern representatives of the lobe-finned lineage. *Latimeria* thus provides a counter-example to the inverse correlation of genetic divergence and frequency of gene birth events apparent for several previously studied chemosensory repertoires of teleosts and tetrapods [8]. Furthermore, the large size of the *Latimeria* T2R repertoire, comparable to some of the smaller olfactory receptor gene families, *cf*. [8], suggests that the sense of taste may require unexpectedly high molecular complexity.

## Methods

### Sequence data mining and phylogenetic analysis

Using representative T2R and V1R amino acid sequences from mouse, *Xenopus tropicalis* and zebrafish as queries, we searched with tblastn for *t2r* and *v1r* genes in the preliminary draft of the *Latimeria chalumnae* genome produced by the Broad Institute [21]. Homology regions above 200 amino acid length were considered further. Several sequences were manually edited to establish or to complete the ORF prediction, including six *t2r* genes, for which ≤2 stop codons/frame shifts were removed, resulting in each case in a full length sequence containing the expected motifs (*cf*. Figure 4) over the entire sequence length. These six genes are indicated with asterisks in the phylogenetic tree (Figure 2). No *t2r* candidate genes with more than 2 stop codons/frame shifts were found. This suggests to us that the edited bases could well have been due to sequencing errors in this draft assembly. One prediction of a small additional N-terminal exon (in V1R10) resulted in lower homology in the multiple sequence alignment, compared to the corresponding full length monoexonic prediction, and so the latter was included in further analysis. Sequences that are >98% identical in amino acid sequence are considered allelic variants [51], but could theoretically result from very recent gene duplications. In this case either adjacent or unambiguously different genomic location would be expected. No such cases were observed. Resulting sequences ranged from 287 to 316 amino acids for T2Rs, and 299 to 321 amino acids for V1Rs. All *Latimeria chalumnae* sequence data used in this article is included in Additional file 2. Sequences were aligned with MAFFT 7 [52], an online version of the multiple alignment tool MAFFT [53], using the E-INS-I strategy with the default parameters. Clustal Omega [54] was also used for alignment.

Phylogenetic analysis was performed with a Maximum likelihood algorithm (PhyML-aLRT) with SPR setting for tree optimization and chi square-based aLRT for branch support [22] on the phylemon2 server [55]. Branch support above 80% was considered significant. Candidate sequences had to fulfil the following stringent conditions to be accepted as *bona fide* unique T2Rs or V1Rs, respectively: a) the gene had to be located inside the corresponding phylogenetic tree with branch support over 80%; b) the sequence had to contain the motifs characteristic for that gene family; c) the sequence had to map to a unique, non-overlapping genomic position; d) the minimally accepted sequence difference of 2% had to be distributed along the sequence.

Sequences were named according to named orthologs or closest paralogs from other species, if applicable, and otherwise according to phylogenetic relationship. The assignment of Lc_V1R04 was confirmed by comparison with V1R-related ORA3 and ORA4-specific motifs.

### Identity and similarity matrices and sequence logos

Pairwise alignments of the 20 V1R and 80 T2R amino acid sequences were performed using the SIAS webserver [56]. Identity and similarity values from all possible comparisons within each family were retrieved and are shown as matrix.

Sequence logos were generated using Sequence logo 3 [26]. Sequence alignments were manually edited using Jalview [57] and positions with gaps in over 90% of sequences were deleted. To align conserved motifs identified within Latimeria T2Rs, V1Rs, tetrapod T2Rs, V1Rs, teleost T2Rs and V1Rs, a multiple alignment including all six gene families was analysed. Transmembrane regions were predicted for multiple aligned sequences using PRALINE [58].

### Analysis of transposable elements

Latimeria scaffolds containing *t2r* and *v1r* gene clusters were examined for repeat elements using RepeatMasker [59], which provides a detailed annotation of class I (retrotransposons) and class II transposable elements. Detailed analysis and graphical representation of results was performed using Excel, Open Office, and Adobe Illustrator. Class I transposable elements (LINE, SINE and LTR) encode a reverse transcriptase (RT) protein enabling a sometimes autonomous “copy and paste” mechanism. Class I elements are most relevant in facilitating gene duplication, inversion and translocation [37–39], and were analysed separately. For reference sequence we randomly chose ten *Latimeria* scaffolds totaling 11.3 Mb genomic sequence.

### dN/dS analysis

The dN/dS ratios for the latimeria *t2r* and *v1r* gene families were calculated using nucleotide sequences aligned by MAFFT [52] and manually edited using Jalview [57] to match the amino acid alignments obtained in phylogenetic analysis. Codon based alignment was also employed using PAL2NAL [60]. To test the selective pressure on individual codons, we used the single likelihood ancestor counting (SLAC) package described in [61] and a fixed effects likelihood (FEL) method that directly estimates nonsynonymous and synonymous substitution rates at each site [33]. As significance cutoff we chose p < 0.1, in accordance with published procedures [33]. To achieve a high stringency of analysis, we required independent prediction of positive or negative selection by both methods. Thus we expect very few false-positives, and indeed no positively selected sites were predicted for several of the gene groups analysed. All dN/dS analyses were performed using the datamonkey server [62]. To exclude saturation bias, we confirmed that dS values for all comparisons were below critical values, *cf*. [32], using DnaSp software package [63].

### Availability of supporting data

The data sets supporting the results of this article are included within the article and its additional files.

## Electronic supplementary material

Additional file 1: **A list of tables containing** ***t2r*** **and** ***v1r*** **gene names, genomic location, previously reported synonyms, homology matrix, detailed motif analysis, and detailed dN/dS analysis.** (XLS 294 KB)

Additional file 2: ***Latimeria chalumnae*** **T2R and V1R protein sequences in fasta format and three phylogenetic tree files in Newick format that were used in construction of phylogenetic trees shown in Figures** 1 **,** 2 **and** 3 **.** (PDF 134 KB)
