# Supplementary material for: Positive Darwinian selection in the singularly large taste receptor gene family of an ‘ancient’ fish, Latimeria chalumnae
Source: BMC Genomics. 2014 Aug 5;15(1):650. doi: 10.1186/1471-2164-15-650 (PMC4132921; doi:10.1186/1471-2164-15-650)
Supplement: Supplementary file 2 — Additional file 2: Latimeria chalumnae T2R and V1R protein sequences in fasta format and three phylogenetic tree files in Newick format that were used in construction of phylogenetic trees shown in Figures 1 , 2 and 3 . (PDF 134 KB) [file 12864_2014_6345_MOESM2_ESM.pdf]

Additional file 2, content:

part 1: Latimeria T2R protein sequences in fasta format. Predicted stop codons in six potential pseudo genes are indicated by X (red background), sequence after frame shift (2 genes) highlighted with yellow color.

part 2: Latimeria V1R protein sequences in fasta format.

part 3: Tree file in Newick format for T2R and V1R sequences shown in Fig. 1.

part 4: Tree file in Newick format for T2R sequences shown in Fig. 2.

part 5: Tree file in Newick format for V1R sequences shown in Fig. 3.

Part 1 **_Latimeria T2R_ protein sequences in fasta format.**

```
>LC_T2R12
MVTVDIILQLAAILFVIVFGIIGNLFIVIINFQELRRTGTLQPSERIVSCIAVSNILAVI
VLAIWFIIFLLDLCTYLGPYIYKVTDFLIVFITKVGYWFTAWLCFFYCVKIVKINWRIFI
KLKQGTSLLVSFLLFATVVCSFGVAYPAIYIIRTLNTTSIVGQCKGYYILGHEFMMGYIV
FVSFIASLLPLALMLLSSLGIVFFLLKHSKNMTKTSNVSSSPQNEGPTTVVKMVISLIVL
YIVSVISILVTNHVATIIESDMVVIIASSSCVFSAGSSVILIVGTVKLRQAFLKIFCSAG
QCYNKCRTK
>LC_T2R11
MVTVDIILQLAAILFVIVFGILGNLFIVIINFQEFRRTGTLQPSERIVSCIAVSNILAVI
VLATWFIIFLLDLCTYLGPYIYKVTDFLIVFITKAGYWFTAWLCFFYCVKIVKVNWRIFM
KLKQGISLLVSFLLFATVLCSFGVAYPVTYVIRTLNTTSIVGECKDYYIIGHEFMMGYIV
FLSVIASLLPLALMLLSSLGIVFFLLKHSKNMTKTSNASSGPRTEGPTAVVKMLISLIVL
YIVSVISILVTNHVATIIESDMVVIIASSSSIFSAGSSVILIVGTVKLRQAFLKIFCSAG
QCCNK
>LC_T2R10
MVTVDIILQLAAILFVIVIGILGNLFIVIINFQEFQRTGTLHPSERIVSCIAVSNILAVI
VLAIWFIIFLLDVCTYLGPHIYKVTDFLIVFITKAGYWFTAWLCFFYCMKIVKINWRIFV
KLKQGISSLVSFLLFATVVCSFGVTYPVIYIIRTLNTTSIVGECKDYYIIGHEFMMGYTV
FLSFIASLLPLALMLLSSLGIVFFLLKHSKNMTKTSSASSGPRTEGPTAVVKMLISLIVL
YIVTVISVPVTNLVATTNESDMVVIIASFSCIFSAGSSVILIVGQVKLRQAFLKIFCSVG
QCCNKCRTK
>LC_T2R09
MVTVDIILQLATILFVIVIGILGNLFIVIINFQEFRRTGALQPSEQIVSCIAVSNILVVV
VLAIWFIIFLLEVCTYLGPHIYKVTDFLIVFFSKAGYWFTAWLCFFYCVKIVKVNWRIFM
KLKQGISSLVSFLLIATTVCSFGVAYPVIDTIRTLNTTNIIGECKDYYILKHEYLVGYII
FLSFIASLLPLALMLLSSLGIVIFLFKHSTNMTKSSNTSSGSRSEGPIAVVKMLISLIIL
YLVSVISVLVTNHVASIIESDMVVIIASSSCVFSAGSSVILIVGTVKLRQAFLKIFCSAG
QCYNKCTG
>LC_T2R07
MATVDIILQLAAILFVIVFGIIGNLFIAIINFQEFWRTGTLQPSERIVSCISVSNILVVI
VLAVWFVIYLLDVCTYLGAHVYKVTDLLIIFISKASYWFTAWLCFFYCMKIIKVKWRFFK
TLKQRISSLVSFLLIATVLCSFAVAYPVIYIIRSLNTTNTIGECKDYYITQHELTMGYAI
FLSLVASLLPLALMLLSSLGIVVFLFKHFKNMTKSSNTSSSPRNEGPITVAKMLISLIVL
YVVTVICVLVTNHVFTIVASDMVVIIASSSCVFSAGSSVILIVGTVKLRQAFLKLFCSVG
RYLQ
>LC_T2R08
```

MATVAIILQLAAIMFVIIFGIIGNLFIVIVNYQKFWRTRTLQPSEQIVSCIAVSNILVVI
VLAVWFTGFLLGVCTYLGAHVYQVTDFLVILFSKSGHWFTAWLCFFYCVKIIKVNWRIFM
KLKQSISSLVSFLLIATVLCNFGVAYPVTYIIRSLNTTNSNEECKDYHIFGHEYLVGYAV
FLSVITSLLPLVLMLISSLGIVVFLLKHSKNMSKTSNTTSGSRSEGPTAVAKMLVSLIIL
YMVSVISALVTNHVATVIQSSMVVIIASSVCIFSAGSSVILIVGTVKLRQAFLRLFCSSV
RCCNKCRA
>LC_T2R25
MATTDAITQLVVELVIMLFGLGGNSFIVCVYVMEYQRSKALLPTEVIVTILAIFNILIQL
NLVLWFVVYLFNLCTHFGEVFYQVTDFNAIFLSKSSYWFTAWLCFFYCVKIVKVNWRVLM
RLKQKMTSVVHILILASLMLSFSVAVPIIYRVKFRKNATSISELCKLYYDTGGESGYIYG
AMMSLLTSFLPLAVMVISSMGIVIFLCRHSRNMTKRGNTGGSSHTDAHTAVALMLLCLIV
LYIICTSTVLSANLQIALSQFDVLLAISFTSSIYSAGSSVILIIGTVKLKQSCGKLCCSG
G
>LC_T2R31
MVAVDAILQMLIAVIIVSISFTGNSFIVHVYFLDYRRNKALKPNELIVTVLAFFNILIQF
NLLLWFMVYLFNLCTFFGDVIYQVLDFSDIFLSESSYWFIAWLCFIYFVKIVKMKRKFFR
SLKQKISLLVNILIFFSMLVNFFLALPVIYMIKLKANSTSLSMLCKDYYITGDTAYIYSA
FLSFFTSFLPLVIMLMSSLGIVIFLCMHSRTMRKNTVAGNSSHGGAHTAVAMMIVCLIVL
YMLCKITVLVANIQIAMAEFDISIAIFCASSIHSAGSSVILIIGTVKLRQSCGTLCCSQN
VSDSNN
>LC_T2R30
MVAVEAILQILTVVIVFISFTGNSFIVHVYFLEYRTNKVLKPNELIVTVFAFFNILIQIN
LLLWFMDYLFNLCTFLGEVIYQVLDLSDIFLTKSSYWFIAWLCFIYFVKIVKIKRKFFKS
LKQKISLLVNILILCSMLVNFFLAVPVIYMIKLKANSTNLSMLCKDYYITGDTIHIYSAF
LSFFTSFLPLVIMLMSSFGIVTFLCMHSRTMRKNTVAGSSSHGDAHTVVTVMIVCLILLY
MLCTVTALAANLQIAMAEFDILVAITFASSIHSAGSSVILIIGTVKLRRSCGALCCSQNV
SD
>LC_T2R32
MVAVDAILQMLIAVIILLISFTGNSFIFHVYFLEYRRNALKPNELIVTVLTFFNILIQFN
LLVWFMVYLFNLCTFLGDVIYQLLDFSDVFLSESSYWFIAWLCFIFFVKIVKIKRKFFKS
LKQKISLLVNILILFTMLVNIFLALPIIYMIKLKVNSTSLSMLCKNYYITEETTHIYSAF
LSFFTSFLPLVIMLMSSLGIVIFLCMHSRTMRKNTVAGSSSHSDAYTAVTMIIVCLIFLY
MLCTATVLTANIQVIMANFDIFVVISFISSIHSAVSSVILITGTVKLRQSCGTLCCPPNV
SDSNK
>LC_T2R26
MVTVDVILQLMAELIIMLIGLAGNSFIFHVYFVEYRRNKALXPTELIVTILAIFNIFIQL
NLVLWFVVYLFNFCTYFGDVIYQVTDFNSIFLSKSSYWFTAWLCFFYCVKIVKVNQRCFR
RLNQRISSLVNILISFSMLISFPVAYPVIYIIKLKVNNTSLSMLCKDYYITGDTTHIYGA
FLSFLTSFSPLAVMLASSMGIVIVLCTHSRNMKRNAVASSSSHGDAHTTVAVMIICLILL
YVLCTITVLSANLQIALSHFNTLIGISFTSSLYSAARSVILIIGTVKLRQSYGTLCCS
>LC_T2R27

MVVIEIALQMLAEVITVLIALAGNLFIVYVYFLEFRKTKALQPNELLVTVLALFNILVQF
SLVLWFMLYLFNLCTYFGGVIYKVTDFNIIFFSKSSYWFTAWLCFFYCMKIIKVKRMFFI
RLKQRMSLLVKILILCTMMLNFSLAYPVVFLIKLKANSTSISILCKDYYITGDTTHIYGA
SLSFLTSLSPLAVMLVSSMGIVIFLCLHSRNMKRNAIAGNSSHGNTHTAVAVMIICLILL
YMLCTITVLSANLQVALAQFDTLVAITFTSSLYSAGSSLILITGTVKLRQSCVTLFCSCR
QQQ
>LC_T2R28
MVAIDAVLQMLAEVIIVLIALGGNSFIFCVYFLDYKRNKTLQPNELLVTFFASFNILIQI
TLIWFVVYLFNLCTYFGEVFYKVMDFSAIFLSKSSFWFIAWLCFIYFLKIVRIKSRFFMG
LQQKMSSLVIVLILITLLVSFSVALPVIYMIKLQTNSTSISELCKDYYIIGKFAYIHGAF
LSILTSFLPLVIMLISSMGIVIFLCTHSRKMKKNAVASSSSNEDAHTAVAVMIICLILLY
VLCIITVLSGNLQIALSQVDETVIIVFMSCIYLAVSPVILIIGTVKLRHSFKKLLTSFKR
YCIKLFNV
>LC_T2R20
MASTNVLLQLVVEFIIVFFGLIGNAFIVHVNLLDYWTRSTLQPSELTVTILALFNIFIQL
NLTFWFIIYLFNLCAYFGDEVYKVTDFLAIFFSKASYWFTAWLCFVYCVKIIKVNWRFFM
RVKKRLNPLVNSLIIGTVLASFAMSFPIVYFIEFKTNSTSISKKCKDYYVDGMNIKIYAA
SLSFLTSFVPLAVMVFSSMGIVIFLYQHSWNKSKNVSSGASSHGDAHTAMAVMLICLNIL
YVACTATVLAANLIIAIVESDIMIAISFTSSIYSAGSSIIIIIGTVKLRQSCGKISLKLL
FCWKQRLM
>LC_T2R21
MASTKVLLQLVVEFIIVFFGLVGNAFIVHVNLLDYWTRSTLQPSELIVTILALFNIFIQL
NLTFWFIIYLFNLCAYFGDEVYKVTDFLAIFFSKASYWFTAWLCFVYCVKIIKVNWRFFM
RVKKRLNPLVNSLIIGTVLASFAMSFPVVYFIEFKTNSTSISKKCKDYYVDGRNIEIYAA
SLSFLTSFVPLAVMVFSSMGIIIFLCRHSWNMSKNVSSGASSHGDAHTAVAVMLICLIVL
YVACTATVLAANLIIAIIESDILIAISFTSSIYSAGSSIIIIIGTVKLRQSCRKL
>LC_T2R22
MAATDVIVQLVVELMIILIGLVGNVFIVVVHVSEYRRTKALQPNELIVAILAFFNILIQI
NLLIWFVVYLLNFCIYFGDDLYKVTDFMSVFLSKSSYWFTAYLCFYYCVKIIKVNWSCFN
SLKQKISSVVKTLITCTLLGTIALSAPVVYYVKLNTNLSSISEICKVYYIDAHNEVYAAF
LSVLTSFLPLAVMFISSTGIVVFLCRHSRNMTKNVTPGGTHNDAHTAVAIMLTCLIVLYI
ACTVTVFAANLLVVTIESDVLVAISFTSSIYSAGSSVILIIGTVKLRKNWVKLCCLGGVI
LQ
>LC_T2R24
MVVDIIILQFVAELFILFFGFLGNFFIVHVYFLEYRKNRALQPTEVMVTFLALFNILIHV
NLVIWFVVYLFNLCIYFDKAFYKVTDVIAIFLSKSSYWFTAWLCFFYCVKIVKVNRKLFV
WLKQRISRMVNTLIIGTMFFSIIISYPCIYVIEIKTNVTSITQRCKDYYITGQSIEIYSP
SLSFLGSFLPLALMVMSSMGIVIFLFRHSRNMTANTTTGSASRNDAHTTVAIMLICLIVL
YLACTVTVFVANIIIAMIANDILIAISFTSSLYATGSSLILIIGTVKLRQSCSKLSCLCR
RCNSKNPQH
>LC_T2R23

MVTVVIVQLLVEFIIMMLGLLGNVFIVYVYFQEYKKSKELQPNELLVAFLAFFNVLIQIN
LGLWFVVYLFNFCIYLGFTDLIALFITNSNYWFIAWLCFFYCVKIIKVNLILFTRLQRRL
PMVVNILIIGTLIGCFPISVPAIHYIKFKTNFTSVSDLCRDYYPHSGNEVYAVLLSVLTS
FLPLVIMVICIMTIVIFLCIHSKNMSKNVTLGGSSHGDAHITVAIMLICLIVFYIACTAT
VFAANLLVVLVDGYVLIAISYTSSIFSTGSAVILISGTVKLKQHFWKICCLRQ
>LC_T2R06
MAGADIIVQVVAVVMITMFGLVGNTFIVLVNLQEFRRSKALQPSERIVTSLALSNGINEI
SQVIWFAVYLMNLCTHTRDDGYKVLDFLAVFISTANYWFTAWLCFFYCVKIVKVNWKSFM
RLKQKISSLVSFLLIGTVLGSFAMSFPIVYYIKIKANTTSLNEKCKDYYIIGNSYHIYSA
FLSFLTCFLPLALMLVSSTGIVVFLCQHSKRMNKSAKAGDGTPSDSHTTVAKMIVSLIVL
YMACVASVLAANHIFTVIESDILVVIAFASSVYSAGSSAILILGTVKLRQSCSKLGCAGM
>LC_T2R13
MAATDDVLQLVAGFILVFLALLGNFFTVLMNLLEFKRSRSLQPNELIMLSLTLSNGLAVI
SYGIWFVIYLMNFCPYFGDTGYQVLDFLSLFLTKTSYWFTAWICCYYCVKVVKVKWRFFM
RLKQRIHKGVNTLIVGTVLGNFVICLPIVFNIKLKTNSTNLKEQCRDYYVLGSSTYIHAT
TLSVLTSFLPLVLMLISSTGIVVFLCKHSRTMNKNSSSGATSHSDAPTAVAKMIVALIIL
YIICTGTVFALSFIVTMVESDTLIVISYMDCLYSAGSSTILIVGTVKLRQSCRRLCCSSQ
LQDY
>LC_T2R05
MAPFAAITQLVMELIIVWIGLAGNLYIMAIYVLEFRKNRTLHPSEIIMTCLASSNTFNEI
SQLVWFLVYLFSLCRHFGDDIYKVLDFLAVFLSAINYWFTAWLSFFYCVKIVKSKWKLFM
RLKQSSSSWVAPVITGTALGCCTISFFIVFYIQIPTNTTNETEPCKDYYIISSDYIIYSA
FYSLLGCFLPLALMAVSSSVIVAFLCKHAKRMRKTTSGASTPQSGTHTAVAKTIFGLIVL
YTSCVVSVLAADHVSILIESDVLIVLAYASSIYSAGSSIILTIGTVKLRRSIRRNCCLKP
SKQRNEVQ
>LC_T2R18
RFELTEEQMANMFLILQLVAVFIVVSLSLLGSFFIVFANLIKFQSSGVLQTGDLIITCLA
LSNRLTDVGQVPWFLVYILNLCNNTGEDLYKAVDFFITFFNKPSSWFAAWLCLIYCVKIV
KVNWRTFLKLDRRISSVVKVLIAVTMVGCLALSLPIIFFIQLQSNTTSISAQCKNYYINE
NSFYVYAAFLSFFTSFLPLAIMLASSMTIVTFLCKHSRKMTNNYNAISGSHGDGPAAVAK
MITSLIVLYILCTGTVFALNNLVVLEGNVLVFIALSCSMYSAGCAMILSINQSINLYL
>LC_T2R19
MANMFLILQLVAVFTVVSLSLLGSFFIVFANLIEFQSSRVLQTGDLIITCLALSNGLTDV
GQVPWFLVYILNLCNNTGEDLYKATDFFITFYNKASSWFTAWLCLFYCVKIVKVNWRTFL
KLKRRISSVVKVLIAVTLVGCLALSLPIIFFIQLQSNTSSISAQCKNYYINENSFYVYAA
FLSFFTSFLPLAIMLASSMTIVTFLCKHSRKMNKSTNAISGSRGDGPAAVAKMITSLIVL
YVLCTGTVFALNSLVVLEGNVLVFIALSCSVYSAGCAMILIIGTVKLKEKCKGVCCRGNR
TEKPKHAVTAASVNR
>LC_T2R16
MANTFLILQLVAIFTVVSLSLLGSFFIVFANLIEFRSSGVLQIGDLIITCLALSNGLTDV
VQVPWFLVYILNLCNNTGEDLYKATDFFFIFFNKASSWFTAWLCLFYCVKIVKVNWRTFL

KLKRRISSVVKVLIAVTLVGCLALSLPIIFFIQLQSNTTSISAQCKNYYINENNIHVYGA
FLSFFTSFLPLAIMLASSMMIVTFLCKHSRKMNKNTNSISVPLRNGPVAVAKVITSLIVL
YVLCTGTVFALNSLVVLEGNVMVFISLSCSVYSAGCSMILIIGTVKLREKCRGVCCRGNR
TEKPKH
>LC_T2R17
MANTFLILQLVAVFTVVSLSLLGSFFIVFANLMEFRSSGVLQTGDLIITCLALSNGLTDV
GQVPWFLVYILNLCNNTGEDLYKATDFLNTFFNKASSWFTAWLCLFYCVKIVKVNWRTFL
KLKRRISSVVKVLIVVTLVGCLALSLPIIFFIQLQSNITSISAQCKNYYINENNSHVYAS
FLSFFTSFLPLAIMLVSCMMIVTFLCKHSRKMSKNTNSISGPLRNGPVAVAKMITSLIVL
YVLCTGTVFALNSLVVLEGNVMVFISLSCSVYSAGCSMILIIGTVKLREKCRGVCCRGNR
TEKPKHS
>LC_T2R14
MANVFLILQLVAVFTVVSLSLLGSFFIVFANLIEFRSRGVLQTGDLIITCLALSNGLIEV
GQVPWFLVYILNLCNNTGEDFYKATDFMSTFFNKASSWFTAWLCLFYCVKIIKVNWRTFL
KLKRRISSVVKVLIVVTLVGCLVVVLPIIFYIQLQSNTTSISAQCKNYYITENNIHVYGA
SLSFFTSFLPLAIMLASSMMIVTFLCKHSRKMSKNTNAVSGSRGDGPAAVAKTITSLTVL
YVVCTGTVFALTSLVVLKDNVLVFIALSCSVYSAGCSMILIVRTVKLKVRCRGLCCRDNR
TEKPKH
>LC_T2R15
MANVFLILQLVAVLTVVSLSLLGSFFIVFANLIEFRSSGVLQTSDLIITCLALSNGLIEV
GQVPWFLVYILNLCNNTGEDFYKATDFLNTFFNKASSWFMAWLCLFYCVKIIKVNWRTFL
KLKRRISSVVKVLIVVTLVGCLVVVLPIIFYIQLQSNTTSISAQCKNYYITENNIHVYGA
FLSFFTSFLPLAIMLASSMMIVTFLCKHSRKMSKNTKAVSRSHGDGPAAVAKTITSLTVL
YVVCTGTVFALNSLVVLEDNVLVFIALSCSVYSAGCSMILIVKTVKLKERCREVCCRGNR
TEKPKH
>LC_T2R34
MAITKVIAELISRLTIIFVGLLGNFFILHIYFLEYRKNKVLHPTELITTLLAFFNLLSSA
ALVLPNLRTLLFCSYFQEPIYKFSDFFSTFFSKSTYWFTACLCFYYCMKIVKVNKRFFLT
LKQRISIVVSVLLLSSFLLCFAVSFPVVYFIQLKPNSSIPCKTHYTLGKALFIYNIFNVA
LAYYLPLVVMMTCSLGIVIFLCRHSRNMDKNIAAGGSSHSDAHKPVAVMLICLILLYMTC
ALTVLLASVQASLGEIDIMTAIPYTASLFNTGSSMILIVGTVKLRQSFRTLWCFCR
>LC_T2R35
MAATKVIVELIIRSTIIFVGLLGNFFILHIYFLEYRKKKVLQPNELITTLLALFNNLSSV
ALVLPAVRTLLFCRYFADPIYKFSDFFSTFFSKSTYWFVACLCFYYCVKIVKVNWRFLLR
LKQRMSTVMNVLLLSSFLLCFAVSVPVAHFIKLKPNSSIPCKTYYTLGKALFIYNIFNVV
LAYYLPLVVMVICSLGIVVFLCHHSRNMDKNVAAGGSSHSEAHKAVAIMLICLILLYMTC
ALTVLLASVQTSLGEIDIMTAIPYTASIFSTGSSMILIIGTVKLRQNFRML
>LC_T2R36
MATTKSLVDLVVRLTIIFVGLVGNAFILHVYSLEYRKSKNLQPNELITALLALFNLLSSA
NLVITTVRTLLFCSYFQEAVYKFTDSFSNFTSKSTYWFTAWLCFYYCVKIVKVNWRFFLR
LKQKISLVVNILLLSTLILCFTISIPIIYLIKLKPNSSVPCKTHFILGRTLFIYGIINGV

LTSYLPLLLMVICSLGIVIFLCQHSRNMDKNVAASGTSHSEAHKAVAIMLISLILLYMTC
AITVLLASVQLTLGEINIMTAIPYTASIFSTGSSVILIIGTVKLRQSFKKSLFFLKTFLY
ESCPF
>LC_T2R37
MPSTSVIVELVVRLTIICVGLVGNTFILHAYFVEYRNRKVLQPNELITTLLALFNLLATA
NLVLPNLRTLLFCSYFGEAIYKFTDSFSNFVSKSTYWFTAWLCFYYCVKIVKVNGRFFLR
LKQRISLVVNILLLSTITLCFTISIPILYLIKLKLNSSVPCKTHFILGRTLFIYGIVNGV
LTSYLPLVVMVICSLGIVIFLCQHSRNMDKSIAAGGTSHSEAHKTVAIMLICLILLYMTC
AITVLLASIQLTLGEINIMTAIPYTASIFSTGSSVILIIGTVKLRQSFKKLWCFCG
>LC_T2R33
MKTTKEIMDLIVRMTIMSLGIIGNFLILHVYFLEYRKSKVLQPNELITALLSFFNLLSSA
AHVLPALRTLVFCTYFKEPVYKFSDFFSFFFSKLTYWYVAWLCFFYCVKIVKVNWKFVLR
LKQRISIVVNILLLGSLILCFALSMPLPYLIKLKANSSVPCRNFYIVGKALFIYGVIIAA
LTAYFPLLIMVISSLGIVIFLCQHSRNMDKNVAAGGSSHSEAHKAVAIMLICLILLYMTC
ALTVLLSSVQVSLGLNDIMTAIPYTATLFSTGSSVILIIGTVKLRQSIKRILCSRC
>LC_T2R39
MATAGTIVEVTLRLVMILFSILWNLFIVRVYFLEYRINKALQPTELIVTLLALFNVCLPV
CLVIPVLRLNFFCRYFGEEVVKLIEVVIIFISKSSYWFTAWLCFFYCMKIVKFNWRFLLR
LKQKISSMVKGLIVFTLMFCFSLSVPIISIIKLKTNSSYTPKECQSIFGRGKSTIIYGVI
FTVFTSLLPLAIMVVSSLTIVIFLCRHSRNLTKNVSTNGSSHSDAHTAVAIMLVCLIVLY
IACTSTVMAANLQASLGNIDLFVGISVSSYLYSGGSAVLLVIGTVKLRQSFKKL
>LC_T2R38
MGTTESIVEVTVRLVIIFFGIIGNLFIMGVYLLKYRRSKALQPTELIVTILAFFNVLIPI
NLVLPMIRGYFLCNYFGEASYKLVDVIVIFVSKASYWFLAWLCFFYCVQIIKVNWKLFLD
LKRKISSVVKVLVFFTLLFCILMSIPVISIIQIQRNSSQVLGHCKNFYMKGNNVFIYGVV
LTALTSLLPLVIMVVSSLSIVIFLCKHSRNMTRNVSTGGSSHSNAHTAVAIMLICLIILF
IVCTFTVIFANLQVSSGEFNILAAISITSSLYSAGSAVILVIGTVKLRKSFKKL
>LC_T2R40
MVTTDAIPELAVRLTLIFFGIIGNLLIVRVYFLEYRISKALQPTELVVTILASINVLQPI
SLFLPIIRLQFFCRYIGEGMVKLIDIILIFFSKMSYWFTAWLCFFYYVQIVKVNWKVFLR
LKQRISLVVKFLIICTLIVCFILSVPVIRIIKFTPNDSYIPKQCQNIYIRGKATFIYTVV
LTVLTSLLPLAVMVVSSLNIVIFLCRHSRNMTKNVTTIGSSHSNAYTTIAIMLISLIILY
VVCTSTVVAVNLQVSVGDLDILTAIAITSSFYSAGSAVILSIGTLKIRQHCIKVCCIKWG
SMLRDQHI
>LC_T2R65
MADNDIVHLCVAMILVVFGCLGNMFILLVYFLEYRRGQTLQPYEVIVTLLSVCCIMIELC
HVVWFPVYLLNFCTNIGDIVYKVTDFINIFLSKTIIWLTAWLCFVYCVKIIKVNQKIFIR
LKKRISLSIRYMIAGTVLLCILLSFPIILFIKLKINSTNICRDYYSVNEKKELSLIYSSM
LTFLTSFLPLVLMLVSSLSIVIFLCQHSRNMDKNVASSSTSHSDAHTSVAIMLICLIALF
IACAGTALSVNIQVATGQFDALEAITLTDIIYSSGSPMILIIGMVKLRNSFVNLLCPMRR
R

>LC_T2R67
EMAANDIVHLCVAMILVGFGCLGNMFILLVFLKEYRRSKTLQPSEVIVTLMSVCCIVTEL
CYALWFPVYMLNFCSYFGDTVYKVTDFINIFLPKTITWLTAWLCFVYCVKIIKVNWRFFM
RLKQRISLVVRYMIVGTLLLCILLGFPIILLIELKVNTTNICRDYYSVNEKKELSLIFSS
MLSVLTSFLPLVLMLVSSLSIVIFLCRHSRNMDKNMASSRTSHSDAHTSVAIMLICLIAL
FVACAGTVLSVDIQVASGQFDVKAVITLVDIIYSSGSPVILIIGMVKLRNSFVNIFKTSI
KTINREGLS
>LC_T2R66
MAANDIVHLCVAMILVGFSFLGNMFIILVFLLEYRRGQTLQPYEVIVILMSVCCIVTDLC
YAVWFPVYLLNICTFFGDTVYKVLDFINTFLPKTLIWLTAWLCFVYCVKIIKVNWRFIMR
LKQRISLAVRYMIVGTLLLCTLLGFPILFFIKLKINSTNICRDYYSVNEKKELSLMYSSM
LSVLTSFLPLVLMLVSSLSIVIFLFQHSRIMNKNVASGSNSHSDAHTSVAIMLLCLIVLF
IACAGTVFSVNIQIATGQFDVLFAITLADIIYSSVSPLILIIGMVKLRNSFVKLLCPLRR
K
>LC_T2R70
MAVTDIVQLGAAIIIIGFGYLGNVFIVLVFLLEYRRNQTLQPHELIVAFMAICNVGTELG
FVVFFVLYLLNLCTYAGETVYEIVHFFTVFLPKTVIWLTAWLCFVYCVKIVKVNWRIFMR
MKQRLFFAVKCMIVGSLLLCTLVSFPVSLLIKFKINSTKICRDYYMSDEEKELFFLFTSI
LSLSTSLLPLILMLVSNLGIVIFLCLHSRAMEKNVSSSGTSHNNAHTSVAIMLLCLIALF
IACAGTALSVNLQIASGQFDVQIAIALSTVIYSSGSPVILLIGTVKLRNSFIILLCPHQR
N
>LC_T2R71
YIIDSIILYLGAAMIIIGFGYLGNAFIILVFLLEYRRSHTLQPHELIVSFMSICNVGTEL
GFVVFFVLYLLNFCTYVGETFYEVVHFFTIFLPKTVIWLTACLCFVYCMKIIKVNWRIFM
RLKQKISLAVNCMIVGTLLLCTLVSFPIALFIKFKINSTNICRDYYTDDEDKEFFFIYAS
SLSLLTSLLPLILMLVSSLGIVIFLCLHSRKMDKNITTNSTSRNDAHTSVAIMLLCLITL
FIACAGTALSVNLQIATGQFDIQIAIALSTVIYSAGSPMILIIGTVKLRNNFTKLLCPNQ
RKISICIFF
>LC_T2R74
MADADIVQLTAAMIIIGFGCIWNVFVDLVFLLEYRRSRTLQPYELIVTLMAIGNIGTEVG
YVIFFVLYLLDLCTYAGETVYKVLQFFTLFCPKIVIWLTAWLCFAYCMKIVKLNWKIFMR
LKLNLSLAVNCMIIGTLLLCFLVSFPVVFFIKFKMNSTNVCRDYYTVEEKREWSFIYTSL
LSFFTSFLPLILMIVSSLGIVIFLCLHSRHMDKNITSSGSSHSDAHTSVAIMLLCLIALF
VVCTGTALSINLQIASGQFDVQVAIALSTVIYSSGSPVILLIGTVKLRNSFFKILCPK

>LC_T2R73
QMVDADIVQLTVAMIIIGFGCIWNVFVDLVFLLEYRRSQTLQPYELIVSLMAVSNIGTEV
GYVIFFVLYLLDLCTYAGETVYKVLHIFTLFFPKTVIWLTAWLCFAYCVKILKVNWKVFM
RLKLRLSLAVNYMIIGTLLLCILMSFPVVFFIKFKINSTNICRDYYTFEKKELSFMYSSL
LSFFTSFLPLILMLVSSLGIVIFLCLHSRNMDKNITSSSSSHSDAHKSVAIMLLCLIALF

IACAGTALSVNLQLATGQFDVQVAIALSTVIYSSGSPVILLIGTVKLRNSFFKFFCPKQR
N
>LC_T2R75
EMADVDIVQLWIAMIIIVFGCLGNTFIILVFSLEYRTSWTLQPYELIVMLMAVCNLGMEL
GYTAFFILYLLDLCTYTGETVYKIIHFFTIFLPKTVIWLTACLCFAYCVKIVKVNCRIFM
RLKQRLSLAVNCMMIGTLLLTILLSFPVILFIKFKMNSTKICRDYYTVNEEKELIFIYTS
LLSFLTSFLPLVLMLVSSLCIVIFLCQHSRYMDKNITPTSTSRSDAHSSVAIMLLCLIAL
FIACAGTALSVNLQIASGQFDVQVAIALSTIIYSSGSPVILLIGTVKLRNSFSKLLSPNK
RNQ
>LC_T2R76
MADADIVQLGVAMIIIVFGCLGNMFITLVFSLEYRRSQTLQPYELIVMLMAVCNLGTELG
YAAFFILYLLDLCTYTGETVYKIIHFFNIFLPKTVIWLTAWLCFVYCMKIVKVNWKIFLR
LKQKLSLVVYCMIIGTLLLTILLSFPVVLFIKLKVNSTNICRDYYTVNEEKELTFIYTSL
LSFLTSFLPLVLMLVSSLGIIIFLCQHTRHMGKNVTPTSTSHSDAHTSVAIMLLCLIALF
IACAGTALSVNLQIATGQFDVQVAIALSTIIYSSGSPVILLIGTVKLRNNFSKLLCPNQK
LLNYMKRL
>LC_T2R68
MVTTDIVQLGVAMIIIGICCLGNAFIILVFLLEYRRSQTLQPYELIVTLMAVSNIGTELD
FVVWFVVYLLHFCTYIGETGYKVTHFFTLFLPKTVIWLTAWLCFVYCVKIVKVNWRIFMR
LKMRLSLAVKCMIIGTLLLCLLLSFPVVFFLKLKMNSTTICRDYYTDEEKKELSFIYTSL
LSFFTSFSPLVLMLVSSLGIVIFLCLHSRNMDTNVTPSGTSHSDAHTSVAIMLLCLIALF
VGCAGIALSVNLQIASGQFDVEIVIALSNIIYSSGSPLILLIGMVKLRKSFTKLLCPNQR
NLSLE
>LC_T2R69
MASTDIVQLGVAMIIIGIGCLGNAFIILVFLLEYRRSQTLQPYEHIITLMAVSNIGTELD
FVVWFIVYLLHFCTYIGETGYKVTHFFTLFLPKTVIWLTAWLCFVYCVKIVKVNWRIFMR
LKMRLSLVVKFMIIGTLLLCLLLSFPVVFFLKLKMNSTTICRDYFTVEEKKEWSFVYTSL
LSFFTSFSPLVLMLVSSLGIVIFLCLHSRNMDTNVTPSGTSHSDAHTSVAIMLLCLIALF
VACAGTALSVNLQIASGQFDVEIVIALSNIIYSSGSPLILLIGMVKLRNSFTKLVCSNQR
NLLLE
>LC_T2R77
EMGTADVVQLGVAMILVGLSCLGNTFIMLVFLMEYRRSRTLQPYELIVTLMAACSIVTVL
AHVVWYIMYLFNFCTYFGSNIYRVTDFINVLLPKTIIWLTAWLCFVYCIKIVKVNWRIFM
RMKKRISLAVKCMITGTLLLCISLSFPSTLFIELKINSTNVCKNYYKADTKNELFFIFTS
MLSLMMSFLPLVLMLVSSLGIVIFLCLHSRNMDKNITSSGTSSCDAHTSVAIMLLCLIAL
FIACAGTVLPVNIQIAFGQFDVLIAIALSNIIYSAGSSVILIIGTVKLRHTLFKLLCQ
>LC_T2R78
EMGAADVVQLGVAMILVGIGCLGNTFIVLVFLLEYRKSQTLQPYELIVTLMAACSVVTVL
VWYVMYLFNFCTYFGSNIYRVTDFINVLLPKTIIWLTAWLCFVYCIKIVKVNWRIFMNMK
KRISLAVKCMIAGTLLLCILLSFPSTLLIELKINSTNVCKNYYKADAKHELFLIFSSMLS
LLTSFLPLVLMLVSSLGIVIFLCLHSRNMGKNITSSGTSRSDAHTSIAIMLLCLISLFIA

CAGIVLCVNLQITSGGQYDLLVAIALSNIIYSAGSSVILIIGTVKPRHTLVKLLCQ
>LC_T2R79
GMATADLVQLGIAMFIIVIGCLGNAYIVLVFLLEYRRSQTLQPYEVIVTLMAACSIVTEL
VSAIWYVVYLLNFCTYFGNPIYKITDFIDIYLPKTIIWLTAWLCFVYCVKIVKVNWRFFM
RLKQRLSLAVKCMIAGTLLLCILLAFPCTLFIRLKLNTTSVCQDYYRADEGKEVFFIYTS
MLSFLTSLLPLILMLFSSLGIVTFLCLHSRNMDKNVTSSSTSRIDAHTSVAIMLLCLIAL
FIACASTALSVNLQVASGQFDVLIAILYTNIIYSSGSPVILVIGTVKLRNIVVKLLCRKQ
G
>LC_T2R72
MADVDIVQLWIAMIIIVFGCLGNTFIILVFSLEYRRSWTLXPYELIVTLMAVYNLDAEFG
YMAFFVLYLLDLCTYTGKTVYKIITVFTVFLPKTVIWLTAWLCFAYCMKIVKLKWKIFMR
LKLNLSLAVNCMIIGTLLCFLLSFPVVFFIKFKMSCTNICRDYYTDEEKKELSFICTSLL
SFFTSFLPLILMIVSSLGIMIFLCLHSRHMDKNITSSGSSHTDAHTSVTIMLLCLIAFFV
VCAGTALSVNFQLASGQFDVQVAIALSTVIYSSGSPVILLNGTVKLRDSFFFKFLCPKQR
N
>LC_T2R57
MEANDIMQMSVSIFILWFGYLGNMFIILVFFLEYRRSRTLQPYELIIMLLALCSMFTEMS
TIIWLIVYFLDLCTYVGEMLYYINDTLITFFPKTAIWFTAWLCFIYCMKIVKLDWKIFMR
LKQKISLAVRFMITWSVLLCAFISIPVSFQIEFTPNITKMCRLYYKSSGQIKFRLIYSSI
LTLLTSLLPLFLMLVSCLGIVIFLCRHSRNMDKNIIPSGTSRNKAHTSVAIMLICLIVLF
VACAGTALSVNTLVASGEFDLAVAIILTQLIYSAGSPVILIIGTVKLRKSFGMLCPSKG
>LC_T2R54
MGASDIMQMSVSMVILWFGFLGNTFIILVFFLEYRRRRTLRPYELIVTLLALCNIFTELS
TVIWLIVYFLDLCTYVGEMLYYINDTLITFFPKTAIWFTAWLCFIYCVKIIKVDWKFFMR
LKQRISLAVNYMITGSVLLCLLLSIPVSFQIEFTPNTTKMCRLYYKPTDDKELRLIYASI
LSFLTSFLPLVLMLASSIGIVIFLCRHSRNMDKNVTPKSSSHSEAHTSVAIMLLCLIAFY
IACAGTALSANLKIVSGEFDLAVAIILTQLIYSAGSPVILIIGTVKLRNSFGKLCFSKR
>LC_T2R56
YYKIVFAFSWSGTNLILQFGFFGNMFIILEYFLEYRRRQTLQSYELIVMLLAFCSIITEL
STVIWCFVYFLDLCTNVGEMLYFVNDSIITFFPKSAIWFTAWLCFVYCVKIVKVNWRFFL
RLKQRISLLVKFMIAWTLLLCFSISIPVSFQIEFTPNITQMCRLYYKSSGKIESRLIYSS
FLSMSTSFLPLVLMLFSSLGIVIFLCRHSRNMDKNINSSSSSRNEAHVSVAIMLLCLIAL
FVACAGTALSANLQIATGEFDLAVAIILTQLIYSAGSPVILITGTVKLRNGFEKRF
>LC_T2R52
VMAPGDIAQLSVAMAILWFGCLGNTFIILVYFLEYRGKRTLQPYEVIVTLLALCSILSEL
DTAVWLLVYFLNLCSYVGEVLYKVTDTLITFFPKSVYWLTAWLCFIYCVIIIRVNWRFFL
RLKQKISLVVNFMILGTLLLCLSISLPVISRIKFRHNSTNMCRQYYIAMDQKEISLLYTS
ILTLLTSFLPLVLMLVSSFGTVIFLCQHSRNMDKNITPTNISHSEAHTSVAIMLICLIAL
FVVCAGTALTINLQVASGQFDLAVAVILTQLIYSAGSPVILVIGTVKLRKYFKKLCSLKS
YCKLHSNTS
>LC_T2R53

MAADDIAQLSVAMAILWFGCLGNTFIILVYFLEYRGKRTLQPYEVIVTLLALCSILSELV
TIVWLLVYFLNLCSYVGEVLYKVTDTLITFFPKSVYWLTAWLCFIYCVIIIRVNWRFFLR
LKQKISLVVNFMIMGTLLLCLSISLPVISRIKFRPNSTNMCRQYYIAMDQKEISLLYTSI
LTLLTSFLPLVLMLVSSLGTVIFLCQHSRNMDKNITPTNISHSEAHTSVAIMLICLIALF
VVCAGTALTINLQVASGQFDLAVATILTQLIYSAGSPVILVIGTVKLRKYFKKLCSL
>LC_T2R43
MIIFGLGCLGNTFIILVFLLEYRRNGMLQPYGLIITLLAVCSSISELAHFTKTVIYVLDF
CAHFLEAVFKLVDFTLVFFSKSSYWLTAWLCFVYCVKIVKIKWRFFMRLKLRIPLVVNFL
IIGTALLCFSISLPVIYRIKFRTNSTYMCKYYYIPMNQKEISLLYTGMLSLLTYFLPLVL
MLVSSIGIVIFLCRHSRNIDKDVTPSDISHSHVHVAVAIMLICLIVLFIVCSTTALSINL
QIASGQLELIIALPLTDLIYSAGSPVILIIGTVKLRKSVGKLHCPKW
>LC_T2R44
KMVAAAILQRYVGMIIFGWGCLGNTFIILVFLLEYRRNGMLQPYELIITLLAVCSSISEL
VHFARAVIYVLDFCAHFLEAVFKLADFTLVFFSKSSYWLTAWLCFVYCVKIVKIKWRFFM
RLKLRIPLVVNFLIIGTALLCFSISLPVIYRIKFRTNSTNMCKHYYITMNQKEISLLYTG
MLSLLTSFLPLLLMLVSSIGIVIFLCRHSRNIDKDVIPSNTSHSHAHVTVAIMLICLIVL
FIVCSTTALSVNLQIASGQLELIIALPLTDLIYSAGSPVILIIGTVKLRKSVGKIHCPKW
>LC_T2R59
MGVADIVQLGVSMILIGIGFYGNTFIILVFLLEYRKSQTLQPYELIVKLMSICSILIESV
YIVKLVVYLLNFCTYFGENIYRVTDFFIILLHKTIIWLNAWLCFIYFVKIVKVNWRIFLR
MKQRISLAVKFMIIGTTLLCFSLSFPVAFRVKFKTNSSSVCRQYFPATDDRKNSFLYSSM
ISILTSFLPLVLMLVSSLGIVIFLCLHSRNMDKNTIPNSMCTSRSDAHTSVAIMLLCLIA
LFIACTGTSLSVNIQVAMGQFEVQIAIALSNIIYLTGSPVILIIGMVKLRNRFAKLICTK
G
>LC_T2R50
MAVSDITLLSVAMFMVWFGCVRNTFVIVVFFLEYRRSRTLQPYEAIIILMALCNLLLVLT
TVSWPVFFFDLCALLGQVFYQVTDTLLIFLPKSIVWLTAWLCFVYCVKIVKVNWRFFLRL
KQKVSLVVKCLITGTMLFCFLLSIPVSFMIKLRPNTTDTCRRYYKATDDIEISLLYTILL
SLLTSFLPLVPMLVSSLGSVIFLCWHSRNTDKNVTPSSTTCSNVHTSIAMMLLCLIAIFI
VCTGTALPVNIQIGIGQHSIRGESAITQLLYSSGSPVILIVILIESRDSRSSASASRRTA
Y
>LC_T2R51
MAISDITLLSVAMVIVWFGCVWNTFVIVVFSLEYRRSRTLQPYEVIIMLMALCNLLVVLI
SASWLIVFFLNLCALLGQVFYQVTDTLLLFLPKSIVWLTAWLCFVYCMKIVKVNWRFFLR
LKQKVSLVVKCMITGTMLFCFLISIPVSFRIKFRPNTTDMCKQYYIATDNLEISLLYTTL
LSLLTSFLPMVLMLVSSLAIVFFLCWHSRSMDKNVTPSSTPHSNAHTSVVVMLLCLIALF
IVCTGTALPVNIQISMGQHSVRGVIAITQLFYSSGSPVILIVGMVKLRKSFMKVCC
>LC_T2R58
VMTTADLVPFLVAMVIIVFGCLGNSFIILVFLLEYKRSQTVQPYELIVILMAICGIVLEL
ISILWFVAYLLNFCTYFGEMIYKVIDFLLILLPKSVIWLTAWLCFVYCVKIVKVNRRFYM
RLKQRLSLAVNFMIAGTALLCISISIPSIFLIKFTTNSTNICRQYYTVKEEQVYSFVFGN

MLSLLTSFLPLVLMVVSSLGIVVFLCQHSRNMDKNVTPTGTSHINAHTSVAIMLICLIVL
FIACAGTVLSLNLQIAAGQLDVLLADSLTNIIYSGGSPVILIIGTVKLRKCLGQLCCP
>LC_T2R60
MATADIVQLCVAVIIIGFACLGNMFIVLVFLLEYRRSQTLQPYELIVTLLAICNLVSEFF
FTLWLVVYLLNLCTYFGEVVYQVTEFIIVFLPKSVIWLTAWLCFVYCVKIIKVNQSLFKR
LKQRISLAVNCMITVTSSVCLLLSFPMVTHIKFRPNSTKICRQYHVVDQELSILYATVLS
LLTSLLPLILMLVSSLGIVIFLCQHSRNMGKNVTPSSTSHSDAHISVAIMLICLIALFIA
CASTILCINITFASGQLDTVLVITLTNIIYSAGSPLILIIGMVKLRHSFAKLFCP
>LC_T2R49
MEVSDIMLLSMGVLIVWFGCIWNMFIILVLTLEYRRSQSLQLYEVIVMLISICNLILEVA
SICWFFVFFLSLCLSVGKLFFQVTETLLLFLPKVVIWLTAWLCFIYCVKIIKVNWGFFLQ
LKQKISLVVKIMATGMVVMCFVISLPVNFIINFRPNTTEVCRKYHMITNNTEMNTIYSTV
ISFFTSLMPLILMLLSCFSIVIFLCQHSRNMDKNINSSSSSRNEAHTAVAIMLFSLTALF
IICAGAALPINIQVGLGQYSTRRIIAITQALYSAGSPVILIMGTVKLRKSFINFCSINHC
KLHSNTL
>LC_T2R47
MTVRDIVFLSVDMTLLWFGSTWNTFIVLAYFLEYRRSQTLQPYEVIVMLIALSNLLMELS
SVSWLVVLFLELCAPLGEVFFQVTDTLLIFFPKSMVWFTASLCCVYCVKIVKVNWRFFLR
LKQKISLFVKFMIFLTLLISISFSLPVYTRIRFNYNSTNMCTHYFFIEGKEQVSIIFAVI
LSFFTSFLPLAIMLVSSLGIVVFLCLHSRNMDKNVTPSSTSRGEAHTSVAIMLLCLIALY
ITCAATAQVVNIEVSSGNFDRREAVAVTELLFAGGSPIILITGIMKLRKVFLKLCFSKGV
KVQT
>LC_T2R48
MTVRDTVFLSVDKTLLWFGSTWNTFIVLAYFLEYRRSQTLQPYEVIIMLIALSNLLMELS
SVSWLVILFLELCAPLERCSSKCFAYLLPKSMVWLTALLCCVYCVKIVKVNWRFFLRLKQ
KISLFVKFMIFLTLLLLISFSLPVHTRIRFNYNSTNMCKHYFIIEGKEQVSVMVAAILSF
FTSFLLLAIMLVSSLCIVVFLCLHSRNIDKNITPSSTSRGEAHTSVAIMLLCRIALYITC
VATAQVVNIEVSSGNFDRREATAVTELLFTGWSPIILIIGIMKLRKVFLKLCFSKGVKVQ
T
>LC_T2R46
MSMRDIVFLSVDMTLTWFSSIWNIFIVLVYFLEYRRSQTLQPYKGIVMLIAFSNLLLELS
TVSSLVVLSLELCAPLGEVFFQVNNTLLIFFPKSMVWLTASLCSVYCVKIIKVNWRFFLW
LKQKTSVFMKFMIFLTLLISSSFSLPVHTRIRFDYNSTNMCKQYYILKGKEEVSVMFTGI
LSFFTCFLPLTIMLVSSLGIVVFLCLHSRNMDKNITPSSTSRVEAHTSVTIMLFCLIALF
VTCAATAQVVNIEISSGNFDRREAITLTELLFAGGSPIILITGTVKLRKVFLKLCFSKGM
KIQT
>LC_T2R45
NMRVTDIVCMSVSMVLLWFGCVWNTFIMVVFFQEYRRTRTLQPYEVIVMLLVVSSILLEL
VSVSWLVIYFLNLCLYVGEVFYQVSDTFILFFPKLIVWFTASLCLVYCVKIVKVNWSFFL
KLKQKISLVVKFMIFLTLLICAFISIPVHSVIKFNFNATNVCREYFILQDETKNSPIYSF
LISTLTSFLPLIIMLISSLGIVIFLFYHLRNMDKNAIPSNTSRNDAHTSVAIMLICLIAL

FVVCAGTALSVNIQIALGQLDIAPAITITQLLYSAGSPVILIVGMVKLRKRFMNLCCSKG

>LC_T2R63
RMGAAAILQMFATMILLGIGCFGNSFIMLVFLVEYKRSWTLQAHELIVTLIVVANIVNEL
IYVFHFAIHFFNLCFYNGETIFALLSLLEGLIPKVISWLTAWLCFVYCVKIIKVNWRFFM
RVKQRISLAVKVMIIGTIGLCCLISVPIYFMIHLKGNTTKGCRDFYVKVSEKELSVLYGG
TLTFFTSFLPLLLMLVSSVSIVIFLCQHSRNMDKNMASSSSSHNEAHTSVAIMLICLIAL
FVVCNGTFLYINVQYAAGHFELLVLSALANTIYSSVSPAILIVGMVKLRHTFAKFFFRKG
RRYSDQ
>LC_T2R64
QNVSRFKLFSTMIIIGFGCLGNSFIVLVFLVEYRRGRTLQAYELIVTLLATCSIVNELLH
VIWFTIYFFNLCFYNGETFFKWFSLLQTFFPKAATWLTAWLCCIYCVKIIKVNWRFFMRL
KQRISLAVKCMIIVTAVLCSLLAMPVLFMVHLKGNSSNNCRELYIQDDEKELSFLFTSML
SLFTSLLPLVFMLVSSLGIVIFLCRHSKNMDKNVTPSSTSRNEAHTSVAIMLLCLIALFI
VCAGTVLFVNLNIAPGRFDLLVVISLSNIIYSSGSPVILIVGMVKLRNTFAKLFCQKPI
>LC_T2R41
MTTSVIVQLVVAMTIMGVGSLGNSFIVLVFFLEYRRFRTLQSYELMVTILAFFSILYEMV
AVLWFVVYWFSFCSYFGEKFYKVTDTLIIFFTKSNYWFTAWLCFVCSMKIIKVKWKLFAR
LKQKISFAVRFMIIGTLLLCFSVSFPIMYGIRFRENSTQMCKNYYIATDKKVNSLAYFGM
LSLMTSFLLLVLMLVSSLGIIIFLCRHSRNISKNVTTSSSSSSNAHSAVAIMLICLIALY
IACTGTALCLNLQVTSGQFDVLIAIAYTNVIYTTVSSVILISETVKLRTSFCRLCCPKTH
K
>LC_T2R42
NMTTAVIVQLVVAMTIMGFGSLGNSFIVLVFFLEYRRFRTLQSYELMVAILAFFSILYEM
VAVLWLVVYWFSFCSYFGETFYKVTDTLIIFFTKSNYWFTAWLCFVYSMKIIKVKWKLFA
RLKQKISFAVRFMIIGTLLLCFSVSFPIVYGIRFRENSTEMCKNYYIATDEKVNSLAYFG
MLSLMTSFLPLVLMLLSSLGIVIFLCRHSRNMSKNVTTSSSSSSDAHLAVAIMLICLIAL
YIACAGTALCVNLQVASGQFNVLIAIAYTNVIYTTVSSVILISGTVKLRTSFCRL
>LC_T2R80
MAIVDIVNLGVAMIIIVISCLGNTFIILVFILEYRRSQMLPYELIITLMAACNLMTELIF
MVWYVVYFFDFCTYLGDPIYKVTDSVTVYVAKTGIWLTACLCFVYCVKIVKVNWRMFMRL
KQKLLVVKYMIVVTLLLCILLAFPVALLIKLNINSTDICWNYYMIEEERELSFLYTGTLS
ILTSFLPLVLMLVSSLGIVIFLCIHSRNMDKNITPGGSTHIKAHTNVAIILLCLIALFVA
CAGTAMSCNLQVVSSQVSVLLAIYYKNIIYSSGSPAILVIGTVKLRDSLVRIFCTKGGGV
TTETVKTLSMCVYNGW
>LC_T2R02
MELSVRVSFLVAFAVAYLLALCMNLFILVRYFHAVRKGEVLQPSDLLLIGLIVCNVVHQT
SILALSVLGLFHVGCYAGGYMFKTLSLVFTSASSAQFWLMAWLCTFYCLNIVRISRRFFI
RMRQCVSGLVPHLLAGSVIGTFAVSLPYFVYVPVVSNNGSLANSTICLMDFRSPAPFYLS
LYLTLNCLLPLVLMVASSSVIIMFLHCHVRRMEENTSGFSSPRSDAYLRITKMLLSLVCL
YISFNVATILMNLIKCNFCGLIISSFAEVYPSLCAIIIILGTSKLRQGSTPHCPRCLCL

```
>LC_T2R03
MNTAYNIGILVIATALTAIGAPGNLFILAINLSTVRSQQVLPPTDLIISGLALSGIVFQT
FLVYLVYMELLGMTCQMEISTFMMIFYITDALGSVNFWFISWLCIFYCAKIVRSGSRLVI
RFQQWINGAVRHLLAWSAVGSFTVPVCYYVFSLGMNHTDILNNCTNLNIAEHKQIVDDSY
YCLYLVLCCLLPLVIMTTSSSFILLFLRRHAMRMQRSAADGFSSPSSEAHVRVSKMVLSL
MGIYTVFDISIMIFIFTSNEILVLITILLCYLCPGVTPVIIIWGTAKLRNRLPTACWSK
>LC_T2R04
MYFDFLIAFCVSIFVLLVIGVSGNLFILVSNLNTVRSQQGLPPSDFILTGLSTCNIIFQI
SNGTIGVLLLLDVIYTMQNYAWRLYFYITELLVSIGFWFTAWLCVFYCAKIIRSSSRLVI
QIQQWIVGAVPHLLVWSAVGNFVVCTPKLVLIFLRNETKTSCNSSFGHFRESKWKLDYMN
TFYLALCCLSPLVIMITSSSFIILFLRRHVAKIRRNTTNGFSSPSSEAYVRVSKMVLSLM
GIYSTFDVCFMISLFTTNEVLFLIIIFLCHVYSSVTAALIILGTTKLRKRFNTVCWSKCC
NLLKIGI
>LC_T2R01
MLPSVQFSLWIINSFIIVASIFGNLFVFLMNYRSYRRKGYFLPCELICCALSAISGFLEV
VFYLWMSMSELDRSCLISQACYISLLLVIFSLHSALLWITAFLLFFYSAKIVIEPIHCYT
KVQDAILKHAPTVLAAIFFFSFTFNIPLIILSKHTANETNTQQCGNLVVRDKIIFLSIYL
PITAILPAVVMVKSSISILVHLMIHLRHLKANTNGFHTPKLSSQMRVVRMTLVLTIVYLI
ALITYVLCMTFAMINNNRLFEIAGTAASIYTMASSLILSYGKQSHWNELAKLWREFVALF
P
>Lc_T2R61
MSAADTVQLGVAVIIIGFGCLGNAFIILVFLLEYRRSRTLQPYEPVVTLVALCSLMAELI
CLVKLVIYMLNLCTYFGETIYKVIDFFTVFLPKSTIWLTAWLCFIYCMKIVKVNWRFFMR
VKQRSTLALKGMIILIAMLCFSLTFPIITHIKFSSNSTNICRQYYIVDQELSFLFASVLS
XLTSLLPLVIMLVSSLGIVIFLCRHSRNIDRNVTPGGTSHSDAHTSVAIMLLCLIALFIA
CAGTAFCVNITLGSGQLDVVIVIALTNIIYSSGSPIILIIGTVKLRNSFAKLFCAKGWTS
SFRQCGC
>Lc_T2R62
MSAADIVQLGVAVIIIGFGCLGNGFIVLVFLLEYRKSWTLQPYESLVTLVALCSLMVELV
YVVKLVIYLLNLCTYFGETIYKVTDFFTVFLPKSTIWLTAWLCFIYCMKIVXVNWRFLMR
VKQRSTLAVKGMITLTALLCFSLTFPIITHIKFSTNICKQYYIVDQELLFLFVSMLSLLT
SLLPLVIMLVSRLGIVIFLCQHSRNIDKNVTPGGTSHSDAHTSVAIMLLCLIPLFIACAG
TAFCVNIMLVSGXLDVVIAIALTNIIYSSGSPIILIIGTVKLRNSFAKLFCAKGLKLSFR
ECGC
>Lc_T2R29
MMAIDAVLQMLAEVIIVLIALAGNSFILCVYFLDYKRNKALQTNELLVTVFASFNILIQI
NLFIWFVVYLFDLCINFGEIFYKVTYFIAIFLSKSSYWFIAWLCFIYFMKIVKIKNRFFM
GLKQNMSLLVIVFILFTFLVSFSVALPGIYMVKLKTNSTSISELCKDXYVIGKFGYIYGA
FLNILTSFLPLVIMLISSIGIVIFLCTHSRKMKKNAVARSSSNEDAHTAVAVMIIYLILL
YVFCTITVLSENLQIALSHVYYVLVTISFISSIYSAGSSVILIIGTVKLRHSFKKLCCLC
>Lc_T2R55
```

```
MGASDIMQMSVSMVMLWFGFLGNTFIILVFFLEYRRSQTLWPYELIVTLLSLCSILMELS
TVIWLIVYFLDLCTYVGEMLYYINDMLIIFFPKMAIWFTTXLCFIYCLKIIKVDWRFFIR
LKQRISLAVNCMITGSVLLCLLLSIPVSFQIEFTPNTTKMCRLYYKPTDDKELRLIYASI
ISLLMLFLPLVLMLASRIGIVIFLCXHSRIMDKNVTPKSSSHSEAHTSVAIMLLCLIAFY
IACAGTALSANLKTVSGEFDLAVAIILMQLIYSAGSPVILIIGMVKLRNSFGKLCFSKR*
```

Part 2 ***Latimeria V1R*** **protein sequences in fasta format.**

```
>Lc-V1R01
MDLCITIKGVSFLLQTGLGVLANLIVLLAYTHIAYSDHKLVAVDMILFHLAFVNMMSLLT
RGIPLTMTIFGLRHILNDAGCVLVVFIYRVVRALSVCITCLLSWFQAITIVPATSKLSRF
KIKVPNYIIPSFVVLWLINIFIYSGVPYYTTAPTKNSSVPKYTMSTGFCYVIFPNQMSFS
LYGVVITSRDLIFVILMILASGYILLILYRHRQQVKALRNPEYSTKSTAESKAAKIVLTL
VLFYIAVFGIENIIGLYVTSLSQVHEYIIDLRVFVSSSYASFSPLIILNFNKKIKSRLRC
AFLE
>Lc-V1R01a
RMDLCTMIKGVSFLLETGLGTLGNFLILLAYAYIVFREHKLSPVDMIFCHLAFANMMVLL
TRGVPQTMTVFGLCNLLNDVECKIVIYTYRIVRALSVCITCLLSMFQAITVAPVTSTWAS
IKMKAPNNIIPSFLALWLINMAVCIAAPFSSKAPRNGTVPEYTLNLGFCHVDFKDQVSYV
INGIAVTMRDCIFVILMVVASCYILLLLHRHSQKVKSIRSSDRNQKTTAETRAAKIVIML
VLLYVVFFGIDNIIWIAMLTIAKVSPVIADMRVFFSSCYAALSPFLIISSNKKIKAVLRC
NSQQREPRDQATDISYVT
>Lc-V1R07
MDLRAIMKATSFLVLTVIGIPGNFTVLAVFSHIAFTEYKLLPTDIIVTNLALVNFILVIS
RGFPQILTAFQLRNLFDTFGCKLIIFAFRIARALSISMTFLLSASQSVTISPATSRLSFL
KQRLPKYLWPLIVFFWLLSGATSVTSILYSTADPNSTASQFTFNLEYCYVAFPGKDAYEG
NGTMYVSRDLTFVILMALASIYILFVLYRHSQQVKSIRNPNRNQGTSAESRAAKTVVTLV
ALYVIFFGVDNLIWIYSISISRVSPMISDIRVFFSSLYATVSPIVIICSNKKIINKLYCT
RRNQVSQAVEIIFTTV
>Lc-V1R11
SMDAYSFLKGVLFLLLAVIGIPCNFAILGAFGKMIYLGKNLFPVEGIICLLALVNTMMIL
TRGVPHILFVFEIRRLYSQHGCAVIIYMARVSRAMAICLTCLLSCSQFLSITPPPSKWIS
LKAILSKTKNLALIVVCLLFLNLGLCVCSVLYAMPETNSTNLNFTYNLGYCIVKFPNRHA
YLGFGLSLLARDLTFVTSMVIASIAIVMTLLRHRQQVSSIRRSSQSHEATVETQAAKSVV
TLVTLYVLFFGIENTIFLYTMTGHQVNSVLSDVRFFFSTCYASVFPIVAIVASSKIREQL
KCFTQSKE
>Lc-V1R12
MNLYNLMKGLIFLLMGIIAIFGNLIIMVLFIHIVYVEGKLMPTEFILLNLAWTNMQMVAS
RGIPQSLYVFGLKKLFNDSGCRAIIYSARISRAMVICLTCHLSCFQCVTIATSNPKWILV
KTKMQKYLVHTIFSLWVFNMFICISRILFTFSPFNSTSPENTFNLGYCVVPFPDKVSFEV
NGYILFTRDIVFVALMALASACILFLLHRHKRQMQSVRRSGRNQESNAENQAAKIVVTLV
SLYVFFFGIETTISLYQTTIPASVVSIVSDVRYFFSICYCTIFPFILCRFNQKIKNKLKS
SVGETEKKQIKAFFCN
>Lc-V1R13
MELYNLMKGIIFFIVAIIGTVGNLVVLALFLHIAYQHKLTAAERLLLNLAWSNSIMLLTR
GVPHSLFVFGLRDLFSDIGCKIVVYLSRVSRAMSICLTCFLSCLQCITIATSTLKWVYYI
KVKMQKYVIPITVFVCLLNMIICIGSVIFSISGTNSNYTQFAFNLGYCIVNFPDKLTLHF
NGFAPFARDIVFVVAMALASAYILLILYRHGKKVKGIRNSEHSHESTAEGQATKTVVTLV
```

TLYIIFFGIDNTIFVYQIAVSKEVHTIVSDIRFFLSICYGLVFPFIIIGFNSKIRTKLKP
SSSEQQTEVQEVSSI
>Lc-V1R14
MDLYNLVKGIIFILTAIVGVSGNLVILVSFYHIALQERKFVTAQVILLNLAWANMIMALT
RGVPHSLFIFGLRFLFDDIGCKIVVFASRVSRAMSICLTCLLSCFQCITITKSTLKWLSL
KGRMQKYVILIIIGLCVMNMLVCIAAVLFSVSSTNTTNLEYTFNLGYCLVTFPDQLSFQV
NGFAIFARDIIFVVLMALASAYILLVLFRHGRQVKGIRSSDRNNVTTAEGQATKTVVTLV
TLYVLFFGIDNTIWFYQITVSKEVHSIVSDIRFFFSVCYASICPIVIIMFNPQIRNKLKA
SSSEQETQVQEISSTQ
>Lc-V1R15
RVIFILITLIGTAGNLVILVSLTHIAYQEHKLLAIEKIVFNLSGANLVISLVRGIPHNLF
LFGFRNLYSDVGCKITGYVHITFRAVAIVLTCLLSCFQCATIARGRSQWTFVKLNLQKHL
EWIIFGLYLFCMISTIDVIPFSISGQNVTNLKFAVSLGYCFVVFPDNIMFQLVGYGIFAR
DFLFVFLMTLASSYILLILYKHRKQVQGIRSTERNHETTAEGQAAKTVVTLVSFYVSVFG
LDNTIWFYEIVSKIMFPVAFDIRNLFSMCYASFFPIVIITFNKKIRHQLKCPRGEQKPQI
ACLSS
>Lc-V1R16
VMDLYEILRRVIFILITLIGTAGNLVILVSLTHIAYQEHKLLAIEKIVFNLSGANLVISL
VRGIPHNLFLFGFRNLYSDVGCKITGYVHITFRAVAIVLTCLLSCLQCATIARGGPQWTF
IKLSLQKHLEWIIFGLYLFCMITTIDVIPLSISGQNVTNFKFAVSLGYCFVVFPDNIMFQ
LVGYVIFTRDFLFMILMTLASSYILLVLYRHGKQVQGIRSTERNHEPTAEGQAAKTVVTL
VSFYVSVFGLDTTIWFYEIVSKRMFPVAFDIRNLFSMCYASFFPIVIITFNKKIRHQLKC
PRGEQK
>Lc-V1R17
KMDPEIVNGAVHFVISIIGIAGNIVILASFSHITYHKRKIMAVEKILVNLSGANLIILVS
RGLPLSLYAFGLRNLFNDFCCQFIGYVHITFRALSVALTCLLSCFQSIMLAKNSPKTAKL
KLKLQTHIVPILSLLCIFSMLCSVDVIVFSVSSYNVTVPQYTAAMGYCLVVFPNIVTFHL
VGYGIFTRDFLFVFLMALASSYILLVLYRHGKQVQGIRSMERNHESTAEGQAAKTVVTLV
SFYVTLFGIDNTIWFYQIISKGLISKVFDIRTLISILYAVVFPFVIIAFNKKIRSKLKCF
TSEQKSEISIIEEMKHIQ
>Lc-V1R18
SMDPETVRGAGYFVITIIGIIGNVVILTSFSHIAYQERKIMAVEKILVNLSGANLIILVT
RGLPLSLYAFGLRNLFNDLCCKIISFVHISFRALSVALTCLLSCFQSIMLAKNSPKTAKL
KVKLQAHIVPILSLLFIFCILCSVDMIVFCISSYNVTVLEYTDTMGYCLEVYPNAVMFHL
VGYGIFARDFLFVFLMALASSYILLVLYRHGKQVQGIRSAEQNRESTAEGQAAKMVVTLV
TFYATLFGIDNIVWFYQTISEHMISQEFDTRTLISLLYAAVFPLVIIVFNKKIQNKIKYF
VSLQKSELSLTE
>Lc-V1R19
MVSVTGRGAVYFVITIIGIIGNVVTLTSFSHIAYQERKIMAVEKILVNLSGANLILLMTE
GLPMSMYSFGLRHCFNDACHEFIYYVKMTFRGLSVVLTCLLSCFQSIMLAPNGPKTAKLK
MKLQVHIVPILSLLFIFCILFNVDMSVFCISSYNITVLEYTDTTEYCFAVMFDLMGYGVF
ARDSLFVFLMALASFYILLVLYRHGKQVQGIRSAEQNRKSTAEGQAAKMVVTLVSFYVTL
FGIDNIVWFYKTFAYQLISQEFDIRMLTSLLYVTLFPFVIVVFNKKIQNKIKYFVSLQKS
ELSLTE
>Lc-V1R08

MEVRLIFKAAGSIFLEALGIPGNALILATFVFIGISNRKLLPADILLMKLAFVNLIVMLT
FNIPTTVSAFGVRKLFNDDGCKTVIFLFRVGRAMSICITSLLSSYQCIVLAPSFNACIIL
KQKCPQSLFHITAFLWCLNTIIYAVCVPSMAEQSFAKSNYSIPMAYCVVSFPSYTFFAVL
GMVFIVRDLLFIAIMVVSSSCIVFILYKHMKQVKGIRSSAKNHGRTAETQAAKAVVMLVI
LYVFLFGLDNIIWAYSLNLSVLVPEITDVRHFLASCFPSISPIIIITTNKKLQNKLRFIS
QRKKLQNAETVVSNVHTI
>Lc-V1R09
MDIQVIFKVISFLLLVVIGIPGNITVMAAFVHLRLSDSKLMPPDIILTKLAFVNLLVVFT
RGVPQVLTALGIKKLFNNNGCRAIIFLFRVSRALSICMTALLSCYQSIVLAPSSKRWRIL
KQKMPQKLLLIVIIFWCVNMLIYSWTLIFSYGPLNSTTEYTLNLEFCFVVFPTFAFYIGN
GTLYLFRDFLFVGLMVLASGYIVFILYQHRKQVKGIRSSDRGQENTAETRAAKAVVMLVA
LYVILFGLDNIIWIYTLQVSKVATVVSDARVFFASCYSALSPILIIATNKKIQMKLKSLG
QNQHHQTPETSVSHVQV
>Lc-V1R10
MNIRVTLKAIGFFLMVVIGIPGNFTILAVFALIKLSNGKLLSTDIILTKLAFVNLLIVLV
KGIPQAFTAIGIRKLFNDNGCRAVLLLYRVSRALSICMTALLSCYQSIIIAPSSNRWRAL
KQKMPQKLVFIMIILWCVNIFIYSWTLSFSFAQLNSTTTEYTLNLEFCFVVFPSFQFYIG
NGTLYLFRDFLFVGLMVLASGYIVFILYQHRKQVKGIRSSDRGQETRAETRAAKAVVMLV
ALYVILFGLDNIIWIYTLQLSKVAPEISDARVFFASCYSAISPILIIATNKKIQMKLKYL
GQKHHHQTPETTISHVQMEM
>Lc-V1R05
MVLSASISEWITYMVFTFIGIVGNSILIHGILTCPTGRLRPSFLLLFSLAAVHMARNVVV
NLLSIIYSAGGVSVFGSAGCKVFKFASALTGTLGIWFTLYTVVFYSVKLEQAVHPLNCAV
NTNWRGYHLAGIFVLWVAGLVVCCPIAVFAEKAKVQVVGNVTHPYRSSVYVGCRCNYPAP
EVALIYGTLALTAIDLVPLVVLAIFSIRIMLLLRKRTGVQFGDIWIGERTETDVFRAAKC
ALLLVLLVTALWVSHFTILQCLRRLDEYYFIPTVLAVLSSGYATLSPYLLMIINYRIRAN
L
>Lc-V1R02
TMGTWSIIKGTAHFILTVIGMPGNLIIVYAFSYAAYSDHKLMPADIIVLNLALVNLMVVL
VRCIPEMLAAYGINELFSDSGCKIVICIYRTTRALSIWLTFLLSGFQCISIAPITTKWAA
FKLQAPGHLFGVLAFLWVFNVGFSIPALLYGISSSGNSTGNGFSINLEYCFVKFPSQYVK
ITVGNLQISRDIIPIFLMIFASVYILLILYHHSQQVKHLQSSNRKRGSSAEIRAAKAVIT
LVLLYVIFFGIDNVLWVYTLTIKAMTTSVISDLRVFFSSLYAAVSPIVIIVSNKKVQKRL
KCEREQQGSLAPETVTSTVRM
>Lc-V1R04
MSQGEHPVQLFFYTMLVALGIVGNSLVIWIVLDTARAANTIPSSDFILLNIAVVNLLISL
TRNTLLLALDIGYTFSMSDGACRILMCIWVWFRCVGVWVTLCLSFFHFMVIRSSHSALGK
INERRNVIVIVAVLWALNLLYSSTALALTSNTSTNISSLVVISSTIRPLLGCVWIFSTDT
AALFYGILSFIVHEIIPIFLMVITNCGTLFLLYKHHRQVHRADIAITRVESEWKAAKTIL
ALILLFVFCWGTHIVSVNYYNFYSSSSTRYMLIIARFSASGFLGFYPLVVTFGHSKLKRK
FHSAVLFWRKQNEVS
>Lc-V1R03
MRFSADNVFYGILVLLGIVGNLLVVVTITVAGYEVGTILASDFILANLAIVNFLISTIRN
VPLFISDLGLKIYLSRDYCKIFMFLWVWLRSVSIWATFCISFFHFLVIRRHHFSVLRKGK
ELRNIIITTSVIWIGNFFYAFPTCFYSTRAYGNETDTIQLLSATTRPFLGCIWKFPSLYS

GVAYATASLVIHEVFPIFLMVVINLGTLYILYRHSRAVGVETLVTRVASERRAAKVILIL
VTLFVICWVTNVLMVNYNHNTEKSIRVFVMLANFGASLFIGFSPVVLMVGHSKLRKKLMN
FTLYLNGIKQKRGKRLTTGK
>Lc-V1R06
MELTRIIFLVIRILVCLFGMTGNVAILSVLIKRAFTFRLKTFEMLLVGLTASNFTQELLV
DVPEIMKELSGVTIHRWFCKTLKFTFTFGRANSIIFTILICIFRYQKLRHAVSRVNLPVP
LDNVKIIHVISASVLAFTFLFSVPVLLHETNVEMTLKNQTSCPALFFDCPKINCEVSYMA
YKLIYLACIDILPILIILVITTHLLRILYKNYKLVSVTLDGFSPNKSKIKDSKVRFWKST
KAVLAALLLFQISWTMQLIIEFAVSSKKFDYWSETDFLIVALYTSLSPYVFGIGNNILT

Part 3 Tree file in Newick format for T2R and V1R sequences shown in Fig. 1.

(Frog_T2R24--Intact_1/1-288:0.0000002748,Frog_T2R25--Intact_1/1-288:0.0061379003,(Frog_T2R46--
Intact_1/1-294:0.3733089728,((Frog_T2R27--Intact_1/1-290:0.2221290239,Frog_T2R28--Intact_1/1-
279:0.4525430920)0.9342195967:0.0616636549,((((Frog_T2R1--Intact_1/1-
289:0.3918210492,Frog_T2R29--Intact_1/10-301:0.3138129339)0.9998500075:0.6509752150,
((Frog_T2R26--Intact_1/1-278:0.3711378041,(Frog_T2R6--Intact_1/1-273:0.0094976265,(Frog_T2R47--
Intact_1/1-273:0.0000002215,Frog_T2R7--Intact_1/1-
273:0.0195082828)0.9763439383:0.0658202988)0.9998500075:0.5006437566)0.9998500075:0.380109
7940,(Frog_T2R31--Intact_1/1-291:0.8850268367,(Frog_T2R48--Intact_1/1-282:0.7657574314,
(Frog_T2R4--Intact_1/1-277:0.6385856814,Frog_T2R5--Intact_1/1-
279:0.4458799738)0.9754412424:0.1318760028)0.9998500075:0.2293353457)0.9865069539:0.123901
5508)0.9378150222:0.0721907075)0.9879578039:0.0652087928,((Mouse_T2R138--Intact_1/1-
294:0.9031576543,(Mouse_T2R137--Intact_1/1-294:0.7042337608,((Mouse_T2R130--Intact_1/1-
291:0.4385864984,(Mouse_T2R106--Intact_1/1-292:0.2818384502,(Mouse_T2R107--Intact_1/1-
291:0.2849591325,Mouse_T2R105--Intact_1/1-
286:0.2660859742)0.9723242732:0.0646695120)0.9998500075:0.3361107536)0.9979043880:0.0868222
191,(Mouse_T2R131--Intact_1/1-284:0.7859857277,(Mouse_T2R122--Intact_1/1-288:0.7037181079,
(((Mouse_T2R110--Intact_1/6-304:0.1082852785,Mouse_T2R123--Intact_1/6-
304:0.1211486462)0.9998500075:0.3223047861,(((Mouse_T2R140--Intact_1/1-
289:0.2436963423,Mouse_T2R33--Intact_1/5-293:0.3706334350)0.9998500075:0.0968005162,
(Mouse_T2R129--Intact_1/1-290:0.4588650720,(Mouse_T2R109--Intact_1/8-
296:0.1985785813,Mouse_T2R117--Intact_1/8-
300:0.1846564081)0.9998500075:0.2022169044)0.9998500075:0.0921111990)0.9998500075:0.0587036
556,(Mouse_T2R116--Intact_1/1-282:0.3428180861,Mouse_T2R115--Intact_1/1-
288:0.4288254583)0.4105026718:0.0346008006)0.9975443582:0.0708063645)0.9998500075:0.132081
8669,((Mouse_T2R102--Intact_1/1-288:0.3628453575,Mouse_T2R121--Intact_1/1-
284:0.2644732676)0.9998500075:0.2189963426,(Mouse_T2R120--Intact_1/1-
277:0.3287041177,Mouse_T2R136--Intact_1/1-
282:0.5241267547)0.9998500075:0.2446176559)0.9414543548:0.0458026860)0.9998500075:0.175700
8610)0.9490440789:0.0649372416)0.9035413101:0.0311880086)0.9632949517:0.0745057136)0.99985
00075:0.2403221472)0.8946574099:0.0910537499,((Mouse_T2R144--Intact_1/1-
296:0.3758235385,Mouse_T2R139--Intact_1/1-296:0.4073920274)0.9998500075:0.4680788472,
((Mouse_T2R118--Intact_1/1-285:0.5965238209,(Mouse_T2R19--Intact_1/1-

285:0.2106430974,Mouse_T2R143--Intact_1/1-
279:0.3094531221)0.9998500075:0.2891300067)0.9998500075:0.2452615405,(Mouse_T2R126--
Intact_1/1-289:0.4058522818,Mouse_T2R135--Intact_1/1-
285:0.6821576804)0.9975668290:0.1547817789)0.9998500075:0.3479581461)0.7979166001:0.073929
7367)0.9998500075:0.0819470797)0.9289610049:0.0581981313,((Frog_T2R30--Intact_1/1-
300:0.9062570021,(Frog_T2R8--Intact_1/1-269:0.9007409140,(Frog_T2R23--Intact_1/1-
271:0.7381519644,Frog_T2R3--Intact_1/1-
275:0.7272333570)0.9987759778:0.2317261906)0.6821274937:0.0912610841)0.9998500075:0.137210
9008,(((((Frog_T2R40--Intact_1/1-289:0.1458985270,(Frog_T2R38--Intact_1/1-288:0.1521294026,
(Frog_T2R22--Intact_1/1-285:0.0345396566,Frog_T2R33--Intact_1/1-
290:0.0428990478)0.9998500075:0.1581041192)0.9961836450:0.0510787711)0.9998500075:0.2797433
222,(Frog_T2R42--Intact_1/1-296:0.3854962307,((Frog_T2R49--Intact_1/1-285:0.0323170027,
(Frog_T2R12--Intact_1/1-287:0.0176918276,Frog_T2R2--Intact_1/1-
296:0.0352917461)0.9998500075:0.0468335502)0.9998500075:0.2154881124,(Frog_T2R36--Intact_1/1-
282:0.1217904578,(Frog_T2R35--Intact_1/1-287:0.1014041177,(Frog_T2R34--Intact_1/1-
278:0.1185097153,Frog_T2R9--Intact_1/1-
287:0.0713945826)0.9998500075:0.0719058910)0.9998500075:0.0414708583)0.9487207693:0.042240
2473)0.9998500075:0.2389850684)0.4102013257:0.0254100847)0.9982245487:0.0767021319,
((Frog_T2R11--Intact_1/1-292:0.1159778516,(Frog_T2R10--Intact_1/1-288:0.0296983799,Frog_T2R32--
Intact_1/1-288:0.0772730699)0.9998500075:0.1086010445)0.9998500075:0.2702914061,(Frog_T2R39--
Intact_1/1-290:0.1469973676,(Frog_T2R37--Intact_1/1-292:0.1459488761,Frog_T2R41--Intact_1/1-
292:0.1553934289)0.9671853200:0.0331965325)0.9998500075:0.2753950401)0.9503958005:0.070057
2092)0.9998500075:0.7052549070,((Frog_T2R43--Intact_1/1-291:0.0578466558,(Frog_T2R44--
Intact_1/1-290:0.0925474181,Frog_T2R45--Intact_1/1-
291:0.1325607965)0.9998500075:0.0700771907)0.9998500075:0.6420054887,((Frog_T2R20--
Intact_1/1-285:0.3373772633,(Frog_T2R17--Intact_1/1-264:0.2007812849,(Frog_T2R13--Intact_1/1-
283:0.1509911882,Frog_T2R15--Intact_1/1-
285:0.1890269594)0.9310123562:0.0516363097)0.9998500075:0.2528057998)0.9998500075:0.492585
9134,((Frog_T2R14--Intact_1/1-298:0.2499519682,(Frog_T2R18--Intact_1/21-
316:0.0458904780,Frog_T2R19--Intact_1/1-
279:0.0331506896)0.9998500075:0.1741341250)0.9998500075:0.1720912984,(Frog_T2R21--Intact_1/1-
294:0.6333956294,Frog_T2R16--Intact_1/1-
288:0.3966415364)0.9998500075:0.2132089124)0.9998500075:0.4025672881)0.9998500075:0.219710
9599)0.7701470506:0.0998139940)0.9047440837:0.0727900033,(((Fugu_T2R2--Intact_1/1-
266:0.5917590332,(Zebrafish_T2R1--Intact_1/1-277:0.0523906386,Zebrafish_T2R2--Intact_1/11-
288:0.1266083844)0.9998500075:0.3121431888)0.9998500075:0.9566320910,((Zebrafish_T2R3--
Intact_1/1-278:0.6843384116,(Zebrafish_T2R4--Intact_1/1-275:0.8506332138,((Fugu_T2R4--Intact_1/1-

265:0.4022612689,Pufferfish_T2R6--Intact_1/1-259:0.2544995359)0.9998500075:0.6254354620,
((Stickleback_T2R1--Intact_1/1-275:0.0525791292,Stickleback_T2R2--Intact_1/1-
271:0.0584899952)0.9998500075:0.5127798477,(Fugu_T2R3--Intact_1/1-265:0.2734646871,
(Pufferfish_T2R3--Intact_1/1-263:0.2071478966,(Pufferfish_T2R4--Intact_1/3-267:0.0793625976,
(Pufferfish_T2R2--Intact_1/3-266:0.0204205591,Pufferfish_T2R5--Intact_1/3-
266:0.0257494947)0.9998500075:0.1229941207)0.9998500075:0.0772810623)0.9998500075:0.182245
6845)0.9998500075:0.1596955346)0.9998500075:0.2215333582)0.8859117687:0.0789688473)0.91883
30177:0.1149542000)0.9998500075:0.8146974982,((LC_T2R01_g03562_fishlike/1-291:0.5112621269,
(Stickleback_T2R3--Intact_1/1-303:0.0800431192,(Fugu_T2R1--Intact_1/1-
302:0.0909182031,Pufferfish_T2R1--Intact_1/1-
302:0.1877375435)0.9998500075:0.1570610348)0.9998500075:0.4874894767)0.9998500075:0.587150
1788,(((Dr_ORA5/1-273:0.3649610913,((Tn_ORA5/1-281:0.0470320901,Tr_ORA5/1-
276:0.0572573062)0.9998500075:0.0497635472,(Ga_ORA5/1-278:0.1135163396,Ol_ORA5/1-
280:0.2697876206)0.9195871606:0.0346282751)0.9987579191:0.1432637128)0.9998500075:0.569521
4806,(LC-V1R06-JH127233-161522-162418/1-265:0.6401632111,(Dr_ORA6/1-266:0.4189193750,
((Tn_ORA6/1-280:0.0807957145,Tr_ORA6/1-280:0.0574373992)0.9998500075:0.1516479691,
(Ga_ORA6/1-282:0.2644439764,Ol_ORA6/1-
266:0.3438426929)0.7466914025:0.0531471395)0.9998500075:0.1973672082)0.9998500075:0.320747
5341)0.9998500075:0.4795662785)0.9998500075:0.3111708831,(LC-V1R05-JH132282-2162-3064/1-
292:1.3014475544,(((Pm_Seq2/1-290:0.4701469976,((Dr_ORA4/5-297:0.1342224155,((Tn_ORA4/5-
299:0.0526967765,Tr_ORA4/5-301:0.0270545865)0.9998500075:0.0727403330,(Ol_ORA4/5-
301:0.1267279037,Ga_ORA4/1-
300:0.1408959339)0.7591087611:0.0142947800)0.9759306732:0.0577260004)0.9998500075:0.4400309
068,(LC-V1R4-JH126562-9351125-9352069/3-289:0.5359678746,((Dr_ORA3/14-308:0.2134264031,
(Ol_ORA3/22-314:0.1474761312,(Ga_ORA3/38-330:0.0919497301,(Tn_ORA3/18-
309:0.0442699298,Tr_ORA3/20-
312:0.0389432232)0.9998500075:0.0669401124)0.9998500075:0.1012879350)0.9976931558:0.0726034
742)0.9998500075:0.3113894861,(LC-V1R03-JH126562-9337639-9338598/1-
297:0.2698094668,Xt_ORA15/4-
295:0.5280105574)0.9998500075:0.1122992802)0.9929369001:0.0718141845)0.6543531594:0.0559505
693)0.9963679795:0.1031874826)0.9998500075:0.3321778687,(Pm_Seq4/4-
290:0.9949707008,Pm_Seq3/29-
284:0.9184710421)0.9368283737:0.1749691667)0.9980564898:0.1359133860,(Pm_Seq1/1-
297:0.9384344551,(((LC-V1R01-JH126576-3892974-3893936/1-303:0.3269506469,(LC-V1R01a-
g18646/2-308:0.1363919186,(Xt_ORA1/1-310:0.3238949512,(Dr_ORA1:0.1390371180,(Ol_ORA1/1-
303:0.0620161150,Ga_ORA1/1-
302:0.0705166541)0.9998500075:0.0710790530)0.9998500075:0.1242877439)0.9918667022:0.052037

8991)0.9998500075:0.0687353780)0.9935904895:0.0803900864,(Xt_ORA14/1-300:0.7036809724,
((((Vmn1r195/1-308:0.2535184127,((Vmn1r216/3-298:0.0814376981,(Vmn1r193/20-
319:0.0518665406,Vmn1r204/20-
319:0.0730623973)0.9998500075:0.0360915502)0.9998500075:0.1336542619,(Vmn1r194/1-
296:0.1939096252,Vmn1r215/1-
300:0.1680779797)0.9998500075:0.0686307341)0.9399434685:0.0247208263)0.9998500075:0.2111152
960,(Vmn1r211/1-298:0.2258934703,(Vmn1r196/1-299:0.2519363793,(Vmn1r200/1-306:0.0804936213,
((Vmn1r206/1-306:0.0365949105,Vmn1r207/1-306:0.0385095472)0.9998500075:0.0581978680,
(Vmn1r208/1-307:0.1346451562,(Vmn1r199/44-349:0.0948531673,(Vmn1r212/1-
298:0.0965359784,Vmn1r222/1-
302:0.1257796968)0.9998500075:0.0490582272)0.9915702152:0.0270576513)0.8331635616:0.014085
0243)0.9335529532:0.0109135184)0.9998500075:0.1196250579)0.9998500075:0.1122326398)0.999850
0075:0.2448738034)0.9998500075:0.3217358136,(((Vmn1r19/1-301:0.0835382723,(Vmn1r18/1-
299:0.0495047902,Vmn1r28/1-
301:0.0383298745)0.9998500075:0.0362496937)0.8546416763:0.0382795064,(Vmn1r25/1-
301:0.1784159592,(((Vmn1r12/1-301:0.0583587722,Vmn1r14/1-
297:0.0429310180)0.9998500075:0.0731126171,(Vmn1r15/1-299:0.1149434140,Vmn1r13/1-
299:0.1070876132)0.9998500075:0.0206831686)0.9998500075:0.0123420514,((Vmn1r24/1-
296:0.0957908258,(Vmn1r26/1-293:0.1001537710,(Vmn1r22/1-301:0.0353979201,Vmn1r23/1-
301:0.0414018059)0.9998500075:0.0804382729)0.9736143814:0.0160659805)0.9998500075:0.034449
8054,(Vmn1r11/1-299:0.0566242484,((Vmn1r32/1-301:0.0234952540,(Vmn1r33/1-301:0.1073045025,
(Vmn1r35/1-296:0.0228412047,Vmn1r36/1-
301:0.0557841404)0.9998500075:0.0446231080)0.9936102676:0.0166012547)0.9998500075:0.050060
1968,((Vmn1r5/1-301:0.0575287419,Vmn1r6/1-301:0.0421086642)0.9998500075:0.0524427340,
(Vmn1r4/1-297:0.1203276224,((Vmn1r16/1-301:0.0520459010,Vmn1r29/1-
301:0.0515510214)0.9998500075:0.0460030266,(((Vmn1r9/1-299:0.0098970805,Vmn1r10/1-
301:0.0094544322)0.9998500075:0.0510150744,(Vmn1r7/1-301:0.0289307896,Vmn1r8/1-
301:0.0108271055)0.9998500075:0.0631200927)0.9998500075:0.0484297703,((Vmn1r20/1-
301:0.0107376297,Vmn1r27/1-301:0.0180417531)0.9998500075:0.0745474642,(Vmn1r17/1-
301:0.0309862887,Vmn1r31/1-
301:0.0588671773)0.9998500075:0.0289385998)0.9998500075:0.0328616990)0.9951658114:0.0190668
313)0.9949974162:0.0124829298)0.9652484268:0.0105926548)0.9998500075:0.0190258743)0.992920
4451:0.0114110023)0.9886157084:0.0105001440)0.9998500075:0.0154816620)0.8932340636:0.013968
4537)0.9978461493:0.0579032907)0.9998500075:0.3640996211,(Vmn1r1/1-304:0.3679369850,
((Vmn1r44/1-308:0.0535305593,((Vmn1r50/1-308:0.1142742902,Vmn1r49/1-
308:0.0671978362)0.9968118880:0.0246717104,(Vmn1r40/1-308:0.0673862500,Vmn1r41/2-
309:0.0872604981)0.9998500075:0.0221614664)0.9852456606:0.0225887811)0.9998500075:0.2235809

830,(Vmn1r54/17-323:0.3475958613,((Vmn1r47/1-308:0.0320841002,Vmn1r48/1-
300:0.0104933483)0.9998500075:0.0411096305,(Vmn1r42/17-324:0.1153081750,Vmn1r45/17-
316:0.0377336451)0.9985729479:0.0259346546)0.9998500075:0.1790324556)0.7767390342:0.029205
9777)0.9998500075:0.1523791491)0.9998500075:0.2426540969)0.9998500075:0.3253116497)0.98180
65400:0.1092591600,((Vmn1r87/1-288:0.4828944499,(Vmn1r85/2-301:0.2928330140,(Vmn1r89/1-
309:0.0746982445,(Vmn1r86/1-309:0.0291067254,Vmn1r88/1-
309:0.1032059548)0.9954634696:0.0272135454)0.9998500075:0.2164004518)0.9998500075:0.143765
9227)0.9998500075:0.3829634951,((Vmn1r172/1-313:0.2137555411,(((Vmn1r56/1-296:0.0450108462,
(Vmn1r57/1-296:0.0350934369,(Vmn1r59/1-296:0.0407726420,(Vmn1r58/1-296:0.0271247706,
((Vmn1r62/1-296:0.0120758336,Vmn1r63/1-296:0.0222052210)0.9856152610:0.0034983179,
(Vmn1r60/1-296:0.0170367987,Vmn1r61/1-
296:0.0530722375)0.9998500075:0.0196012873)0.9998500075:0.0150310534)0.9998500075:0.045006
2711)0.9647578952:0.0092821054)0.9998500075:0.0260535361)0.9998500075:0.2115403845,
(Vmn1r171/13-316:0.0073850505,(Vmn1r176/1-304:0.0692301808,Vmn1r175/1-
304:0.0250728337)0.9024098068:0.0206400442)0.9998500075:0.1562476135)0.8594804693:0.032955
4522,((Vmn1r90/1-309:0.0413912181,(Vmn1r168/1-309:0.0153415262,Vmn1r177/1-
309:0.0120307345)0.9998500075:0.0373887750)0.9998500075:0.1782642326,(Vmn1r178/1-
304:0.0965793663,((Vmn1r181/1-304:0.0533991160,Vmn1r182/1-
305:0.0924727105)0.9998500075:0.0482845715,((Vmn1r102/1-313:0.0090525495,(Vmn1r93/1-
313:0.0000001141,Vmn1r126/1-
313:0.0106454392)0.9998500075:0.0070162165)0.9998500075:0.0713590280,((((Vmn1r98/1-
307:0.0054474407,Vmn1r119/1-307:0.0054408523)0.9943159346:0.0030313337,(Vmn1r101/1-
307:0.0245356007,Vmn1r92/1-
307:0.0083656529)0.9998500075:0.0081955843)0.9998500075:0.0103530367,(Vmn1r105/1-
307:0.0180238573,(Vmn1r96/1-307:0.0146237894,Vmn1r129/1-
307:0.0155265248)0.6431292273:0.0047410986)0.9998500075:0.0222575235)0.9998500075:0.048809
7658,(((Vmn1r94/1-305:0.0110527150,Vmn1r127/1-305:0.0082112562)0.9659020594:0.0027389874,
(Vmn1r103/1-305:0.0166547418,(Vmn1r99/1-305:0.0028097181,Vmn1r120/1-
305:0.0193618595)0.9998500075:0.0223051506)0.9998500075:0.0084387637)0.9998500075:0.022984
9297,(Vmn1r123/1-307:0.0354905487,(((Vmn1r109/1-297:0.0199108126,(Vmn1r100/1-
307:0.0032567378,(Vmn1r125/1-307:0.0087014451,(Vmn1r128/1-307:0.0164217451,(Vmn1r95/1-
307:0.0027174720,Vmn1r104/1-
307:0.0108821596)0.9790247256:0.0026942667)0.9998500075:0.0159186680)0.9974158484:0.005439
2496)0.9998500075:0.0049070602)0.9877068075:0.0030420996,(Vmn1r91/1-
307:0.0167669732,Vmn1r124/1-
307:0.0303099935)0.9998500075:0.0109573762)0.9998500075:0.0180806942,(Vmn1r106/1-
306:0.0256004032,(Vmn1r97/1-306:0.0034836168,Vmn1r108/1-

306:0.0074668079)0.9998500075:0.0240500920)0.9998500075:0.0211584355)0.9998500075:0.0101404
780)0.9837921156:0.0090363142)0.9998500075:0.0145877047)0.9998500075:0.0244384241)0.999850
0075:0.0262089835)0.1964229356:0.0137875032)0.9998500075:0.1685808970)0.9344788967:0.04144
58475)0.9958328581:0.0762571595)0.9998500075:0.8122053460,((Vmn1r82/1-304:0.2346002096,
(Vmn1r84/13-318:0.3404437137,(Vmn1r78/3-310:0.1514934665,((Vmn1r81/3-
306:0.0987251292,Vmn1r83/3-306:0.0744502212)0.9998500075:0.0589657392,((Vmn1r75/3-
305:0.1379910580,(Vmn1r73/1-303:0.1726011144,(Vmn1r76/21-324:0.0973240785,(Vmn1r74/1-
304:0.1152668020,Vmn1r80/1-
308:0.0737085091)0.4727186922:0.0097204351)0.9998500075:0.0552372772)0.6567833024:0.006291
4913)0.7867835858:0.0173923993,(Vmn1r77/3-306:0.1880005519,(Vmn1r79/3-306:0.0000000001,
(Vmn1r2/3-306:0.0141274731,Vmn1r3/3-
306:0.0084845421)0.9998500075:0.0056334144)0.9998500075:0.1223774570)0.9998500075:0.065508
0654)0.9998500075:0.0277914315)0.9990517648:0.0352098535)0.9998500075:0.1625338603)0.99802
46235:0.0607966432)0.9998500075:0.2821296737,(((Vmn1r227/1-306:0.2189809497,(Vmn1r228/31-
333:0.1441562138,Vmn1r232/46-
351:0.1291740381)0.9672893531:0.0414372148)0.9998500075:0.1884860397,(Vmn1r224/1-
298:0.3242896062,((Vmn1r66/6-310:0.1965625953,(Vmn1r67/1-305:0.1648433886,(Vmn1r68/15-
319:0.0683106813,Vmn1r69/15-
319:0.0545759492)0.9998500075:0.1147077739)0.9778973356:0.0262565747)0.9998500075:0.0412009
514,(Vmn1r71/1-307:0.2170029431,(Vmn1r184/5-312:0.0564783864,Vmn1r185/6-
313:0.0409568211)0.9998500075:0.1837617072)0.9998500075:0.0708771047)0.9998500075:0.1363668
492)0.6382194316:0.0314257888)0.9998500075:0.1823405606,(Vmn1r234/24-
329:0.4202983084,Vmn1r237/1-
289:0.2622186627)0.9998500075:0.1278761111)0.9998500075:0.1695444723)0.9536314793:0.0946716
591)0.9998500075:0.1749320407)0.8827857700:0.0976590235)0.9998500075:0.2571863956)0.999850
0075:0.1971083531)0.9998500075:0.0824612660,((LC-V1R11-g13453/2-306:0.5094854109,(LC-V1R12-
JH127167-62652-63599/1-307:0.3543450500,((LC-V1R13-g01824/1-307:0.2098836459,LC-V1R14-
g02196/1-307:0.1452640638)0.9451573690:0.0254390077,((LC-V1R15-JH132386-1786-2700/1-
299:0.0187769964,LC-V1R16-g00870/2-306:0.0353416568)0.9998500075:0.2231965550,(LC-V1R17-
g00958/2-306:0.0635069471,(LC-V1R18-g00627/2-306:0.0252269692,LC-V1R19-JH129249-102462-
103379/1-
300:0.1624446306)0.9998500075:0.1007316348)0.9998500075:0.1936552369)0.9998500075:0.256006
7997)0.9991481052:0.0590118198)0.9998500075:0.0879918245)0.9998500075:0.1256091397,(((LC-
V1R08-JH126576-3899564-3900517/1-305:0.4309083460,(LC-V1R09-JH126576-3966530-3967480/1-
305:0.0936824172,LC-V1R10_JH126576-3956453-3957412/1-
306:0.0980134093)0.9998500075:0.1391193264)0.9815662521:0.0535146681,(Xt_ORA12/1-
308:0.3870049181,(Xt_ORA11/1-203:0.4741998587,((Xt_ORA10/1-306:0.0976172529,(Xt_ORA9/1-

309:0.0667287639,Xt_ORA8/2-
307:0.0536666318)0.9944189458:0.0317394900)0.9998500075:0.1566216355,(Xt_ORA7/1-
303:0.2145375564,(Xt_ORA13/1-310:0.4292760553,(Xt_ORA6/16-321:0.2255586176,(Xt_ORA2/1-
306:0.0000006663,(Xt_ORA5/100-405:0.0423263167,(Xt_ORA3/1-283:0.0093074736,Xt_ORA4/1-
306:0.0473048831)0.9998500075:0.0168786753)0.9998500075:0.0359822185)0.9998500075:0.157183
8228)0.9998500075:0.0573460003)0.9955751782:0.0271449577)0.9990945378:0.0372537859)0.69493
84880:0.0721555293)0.9998500075:0.1134169091)0.9998500075:0.1567042044)0.9998500075:0.18569
71104,(LC-V1R07-g18654/1-307:0.3024141840,(LC-V1R02-g18669/2-309:0.2610135486,(Dr_ORA2/1-
301:0.3567305545,(Ol_ORA2:0.2311281364,(Ga_ORA2/1-305:0.1417865184,
(Tn_ORA2:0.0913400963,Tr_ORA2:0.0464929181)0.9998500075:0.1155857952)0.9051379923:0.03875
94650)0.7319420337:0.0483282812)0.9998500075:0.3654060128)0.9998500075:0.1724694439)0.9931
618418:0.0637175510)0.9998500075:0.0644630965)0.9434175942:0.0602583371)0.9998500075:0.390
0067360)0.9998500075:0.2390912256)0.8507682672:0.0926918176)0.8586983343:0.1354216159)0.99
98500075:0.3914441513)0.4219211871:0.0848933637)0.8771592670:0.0781197942)0.9497968092:0.07
16360678,((LC_T2R02_JH128442-389500-390500/1-290:0.5438420463,
(LC_T2R03_ENSLACG00000000459/5-293:0.3606516550,LC_T2R04_ENSLACG00000005021/1-
297:0.3297282562)0.9998500075:0.3507349448)0.9998500075:0.2641769056,
((LC_T2R07_ENSLACG00000000429/1-296:0.0874511966,(LC_T2R08_JH130784-23237-24160/1-
300:0.1393603167,(LC_T2R09_ENSLACG00000003709/9-308:0.0657440966,
(LC_T2R10_ENSLACG00000002588/1-301:0.0461297376,(LC_T2R12_JH130784-b58450-b59400/4-
304:0.0482327009,LC_T2R11_ENSLACG00000000516/1-
297:0.0187790059)0.9998500075:0.0113812452)0.9998500075:0.0385968711)0.9998500075:0.0367074
197)0.9938009396:0.0281784928)0.9998500075:0.2650220881,
(((LC_T2R06_ENSLACG00000000717/1-298:0.1729165304,LC_T2R05_ENSLACG00000002015/1-
302:0.3884434864)0.9998500075:0.0739596212,(LC_T2R13_JH130976-3613-4524/1-
301:0.3079661889,((LC_T2R14_g02292/13-312:0.0297769995,LC_T2R15_JH129824-107559-108476/1-
300:0.0191366820)0.9998500075:0.0435393963,((LC_T2R16_g02330/13-
312:0.0263963155,LC_T2R17_g02899/1-300:0.0231022472)0.9998500075:0.0254163739,
(LC_T2R18_JH131589-29724-30617/9-295:0.0697563605,LC_T2R19-124199-125143/1-
300:0.0158576479)0.9998500075:0.0286694575)0.4975738928:0.0162428475)0.9998500075:0.368162
3688)0.9946076484:0.0406660497)0.8336642436:0.0410956625,((LC_T2R20_g00491/11-
312:0.0491719541,LC_T2R21_g05130/13-305:0.0025899078)0.9998500075:0.1716038016,
((LC_T2R22_JH129648-127088-127993/1-298:0.1826205054,LC_T2R23_ENSLACG00000001021/1-
291:0.2776269152)0.9998500075:0.0738591837,(LC_T2R24_ENSLACG00000001744/4-
304:0.2578330282,((LC_T2R25_JH129648-116200-117050/1-299:0.1570825098,
(LC_T2R26_JH130917-46105-46999/1-296:0.1061386018,(LC_T2R27_ENSLACG00000001874/1-
301:0.1769913626,((LC_T2R28_g06895/1-301:0.0905853542,Lc_T2R29_JH128085-30766-31662/1-

296:0.0796232509)0.9998500075:0.1392743943,(LC_T2R30_ENSLACG00000001246/1-
300:0.0759321856,(LC_T2R31_JH128085-329000-329900/1-303:0.0481266071,LC_T2R32_JH128085-
412667-413584/1-
302:0.0930365250)0.8332724751:0.0134221100)0.9998500075:0.1299614931)0.9998500075:0.0449467
295)0.9998500075:0.0414526907)0.9998500075:0.1090341758)0.9985025540:0.0480894639,
(((LC_T2R33_g01751/1-294:0.1602040477,((LC_T2R34_JH129988-117000-119000/1-
295:0.0815883961,LC_T2R35_ENSLACG00000000678/13-
302:0.0717128781)0.9998500075:0.0754846922,(LC_T2R36_g01331/1-
296:0.0672338653,LC_T2R37_JH129988-167404-168291/1-
295:0.0747134713)0.9998500075:0.0722779241)0.9880052695:0.0350972021)0.9998500075:0.222578
4555,(LC_T2R38_ENSLACG00000000317/13-304:0.1945327194,(LC_T2R39_g01372/11-
302:0.2115636487,LC_T2R40_ENSLACG00000002445/1-
301:0.2157127117)0.9992448797:0.0422334636)0.9998500075:0.1161984128)0.9998500075:0.0980602
648,((LC_T2R41_JH130077-122561-123463/1-
298:0.0455205165,LC_T2R42_ENSLACG00000006135/4-
294:0.0159460994)0.9998500075:0.1948524874,((LC_T2R43_JH132281-3349-42091/1-
284:0.0427961296,LC_T2R44_ENSLACG00000005213/2-
297:0.0161055169)0.9998500075:0.2264536339,((((LC_T2R45_ENSLACG00000002953/2-
297:0.1924104697,(LC_T2R46_JH132617-3356-4267/1-301:0.1011351041,(LC_T2R47_JH134280-
2000-3700/1-301:0.0192549555,LC_T2R48_JH131192-13968-14870/1-
298:0.0877459416)0.9998500075:0.0382600588)0.9998500075:0.2228467368)0.9998500075:0.060716
6728,(LC_T2R49_g03496/1-298:0.3550123034,(LC_T2R50_JH135454-2600-3600/1-
298:0.1593226990,LC_T2R51_JH130976-56756-57640/1-
293:0.0513476091)0.9998500075:0.0873911273)0.9990684779:0.0569047827)0.9998500075:0.0988790
491,((LC_T2R52_ENSLACG00000002071/2-300:0.0238464334,LC_T2R53_ENSLACG00000002046/1-
294:0.0014817312)0.9998500075:0.1178236468,((LC_T2R54_JH130641-11547-12443/1-
296:0.0111576799,Lc_T2R55_JH130827-7169-8065/1-294:0.0650336000)0.9998500075:0.0639817088,
(LC_T2R56_ENSLACG00000001061/14-305:0.2001696710,LC_T2R57-AFYH01284138-450-1400/1-
296:0.1060967258)0.9998500075:0.0356116258)0.9998500075:0.1244549694)0.8636328556:0.0243862
984)0.9998500075:0.0817106275,(LC_T2R58_g06318/2-295:0.1982464456,
((LC_T2R63_ENSLACG00000002255/3-302:0.2805577997,LC_T2R64_ENSLACG00000003395/1-
296:0.1325898332)0.9998500075:0.1856375380,((LC_T2R60_JH128916-233505-234389/1-
294:0.1774338232,(Lc_T2R61_JH128916-195708-196628/1-300:0.0557028439,Lc_T2R62_JH129837-
106889-107800/1-297:0.0470603442)0.9998500075:0.1073746441)0.9998500075:0.0635388340,
(LC_T2R59_JH128916-126493-127395/1-296:0.1988111591,(((LC_T2R68_JH129750-41217-42131/1-
300:0.0147140432,LC_T2R69_ENSLACG00000003809/1-
300:0.0351695026)0.9998500075:0.0683722006,((LC_T2R70_JH129303-210000-211000/1-

298:0.1007410726,LC_T2R71_ENSLACG00000003104/2-
301:0.1087302132)0.9998500075:0.0729803060,((LC_T2R72_JH129303-64562-65465/1-
297:0.1046414724,(LC_T2R73_JH129303-155997-156905/4-301:0.0563682333,LC_T2R74_g01418/1-
295:0.0574331928)0.9944099307:0.0391532156)0.9921407445:0.0453540276,(LC_T2R75_JH129303-
138585-139493/2-300:0.0655458514,LC_T2R76_ENSLACG00000007023/1308/1-
300:0.0583549065)0.9998500075:0.0390685899)0.8636269225:0.0223374627)0.9998500075:0.034318
1311)0.9998500075:0.0500044719,((LC_T2R65_JH131022_JH1310226900-7950/1-298:0.0866603704,
(LC_T2R66_JH128085-553378-554280/1-298:0.0880937982,LC_T2R67_ENSLACG00000002847/21-
321:0.0982410740)0.9579907740:0.0145478743)0.9998500075:0.1030665200,((LC_T2R77_g03692/2-
295:0.0482303432,LC_T2R78_g07306/2-292:0.0601651143)0.9998500075:0.1117312779,
(LC_T2R79_ENSLACG00000003761/4-300:0.1090817943,LC_T2R80_JH128916-42068-43018/1-
298:0.2561175850)0.9998500075:0.0350922020)0.9998500075:0.0348920757)0.9929746850:0.0229672
452)0.9998500075:0.0378201760)0.9921686801:0.0224180492)0.2139784204:0.0107735547)0.999850
0075:0.0412249322)0.9985106044:0.0372263032)0.9998500075:0.0258295111)0.9998500075:0.069585
9488)0.9998500075:0.1886715326)0.9998500075:0.0357617226)0.9998500075:0.0398151067)0.43350
41674:0.0271590727)0.9998500075:0.0401050970)0.9998500075:0.0847427775)0.9998500075:0.1098
686526)0.9998500075:0.4153742365)0.9039926935:0.0985613767)0.9998500075:0.2380142825)0.998
7257097:0.0827871811)0.9998500075:0.1527602807)0.9998500075:0.9337756594)0.6950113924:0.052
6770126)0.9998500075:0.3822693929);

Part 4 Tree file in Newick format for T2R and V1R sequences shown in Fig. 2.

(Frog_T2R24--Intact_1/1-264:0.0000002264,Frog_T2R25--Intact_1/1-264:0.0033918876,(Frog_T2R46--
Intact_1/1-265:0.3244729492,((Frog_T2R27--Intact_1/1-266:0.2599625400,Frog_T2R28--Intact_1/1-
257:0.4376057685)0.8870291968:0.0625733451,((((Mouse_T2R138--Intact_1/1-287:0.8612148069,
(((Mouse_T2R115--Intact_1/1-281:0.4150458937,((Mouse_T2R110--Intact_1/6-
297:0.0987915022,Mouse_T2R123--Intact_1/6-297:0.1048446294)0.9998500075:0.2998708539,
(Mouse_T2R116--Intact_1/1-283:0.3544824036,((Mouse_T2R140--Intact_1/1-
284:0.2265421711,Mouse_T2R33--Intact_1/1-285:0.3567211527)0.9998500075:0.1044054501,
(Mouse_T2R129--Intact_1/1-279:0.4230826603,(Mouse_T2R109--Intact_1/1-
284:0.1622910546,Mouse_T2R117--Intact_1/1-
285:0.1910237582)0.9998500075:0.2045263092)0.9998500075:0.1007780975)0.9804359879:0.052413
4258)0.9296416960:0.0455409258)0.9473144087:0.0604756598)0.9998500075:0.0996017303,
((Mouse_T2R102--Intact_1/1-282:0.3658903576,Mouse_T2R121--Intact_1/1-
277:0.2531435025)0.9998500075:0.1945241555,(Mouse_T2R120--Intact_1/1-
270:0.3122862773,Mouse_T2R136--Intact_1/1-
282:0.5108217758)0.9998500075:0.2574453838)0.9117966713:0.0609282666)0.9998500075:0.1612137
430,(Mouse_T2R131--Intact_1/1-276:0.7555573328,((Mouse_T2R130--Intact_1/1-282:0.4268849725,
(Mouse_T2R106--Intact_1/1-285:0.2887544561,(Mouse_T2R107--Intact_1/1-
283:0.2835983267,Mouse_T2R105--Intact_1/1-
278:0.2522530118)0.9916686581:0.0692464803)0.9998500075:0.3343427037)0.9369605795:0.0521811
097,(Mouse_T2R137--Intact_1/1-283:0.5823961833,Mouse_T2R122--Intact_1/1-
280:0.6326662260)0.9387415686:0.0979965175)0.9822908464:0.0478860042)0.7210801157:0.0564103
504)0.9998500075:0.2470113286)0.9974379881:0.1052243949,((Mouse_T2R144--Intact_1/1-
273:0.4106612910,Mouse_T2R139--Intact_1/1-269:0.4481781038)0.9998500075:0.3418388627,
((Mouse_T2R118--Intact_1/1-272:0.6615004244,(Mouse_T2R19--Intact_1/1-
271:0.2155900159,Mouse_T2R143--Intact_1/1-
272:0.2751339249)0.9998500075:0.2530864526)0.9998500075:0.2238310610,(Mouse_T2R126--
Intact_1/1-279:0.4288574979,Mouse_T2R135--Intact_1/1-
275:0.7656949928)0.9998500075:0.1724371662)0.9998500075:0.3089406733)0.9906939019:0.093093
9927)0.9998500075:0.1282194928,((Frog_T2R1--Intact_1/1-287:0.3510779989,Frog_T2R29--
Intact_1/10-284:0.3183371215)0.9998500075:0.5895760922,((Frog_T2R26--Intact_1/1-
277:0.3608410142,(Frog_T2R7--Intact_1/1-269:0.0000028659,(Frog_T2R47--Intact_1/1-
269:0.0000002107,Frog_T2R6--Intact_1/1-
269:0.0925107661)0.9329005514:0.0191467137)0.9998500075:0.5181170213)0.9998500075:0.2971507

864,(Frog_T2R31--Intact_1/1-268:0.9432331625,(Frog_T2R4--Intact_1/1-264:0.5292968107,
(Frog_T2R5--Intact_1/2-276:0.4452445211,Frog_T2R48--Intact_1/1-
276:0.7105934744)0.9793655660:0.1044632915)0.9998500075:0.3252907061)0.6563109063:0.051033
3140)0.9998500075:0.1117206212)0.8673286692:0.0296372284)0.8137837545:0.0554855079,
((Frog_T2R30--Intact_1/1-282:0.8272648301,(Frog_T2R8--Intact_1/1-264:1.0199302889,(Frog_T2R23--
Intact_1/1-262:0.6899544419,Frog_T2R3--Intact_1/1-
276:0.7798721483)0.9985338407:0.1952288827)0.9072052983:0.0973268139)0.9998500075:0.173352
5408,((((((Frog_T2R49--Intact_1/1-281:0.0299816200,(Frog_T2R12--Intact_1/1-
283:0.0173398816,Frog_T2R2--Intact_1/1-
292:0.0351097668)0.9998500075:0.0377564572)0.9998500075:0.2187163195,(Frog_T2R36--Intact_1/1-
280:0.1157335647,(Frog_T2R35--Intact_1/1-283:0.0920338546,(Frog_T2R34--Intact_1/1-
274:0.1182698262,Frog_T2R9--Intact_1/1-
283:0.0705768125)0.9998500075:0.0670300030)0.9998500075:0.0425922766)0.9068766681:0.036843
4662)0.9998500075:0.2424215387,(Frog_T2R42--Intact_1/1-291:0.3789200934,(Frog_T2R40--
Intact_1/1-284:0.1387220718,(Frog_T2R38--Intact_1/1-283:0.1470968205,(Frog_T2R22--Intact_1/1-
283:0.0499817398,Frog_T2R33--Intact_1/1-
285:0.0437672686)0.9998500075:0.1442197664)0.9998500075:0.0506431638)0.9998500075:0.271623
1439)0.3489861944:0.0160885373)0.9998500075:0.0795845681,((Frog_T2R11--Intact_1/1-
288:0.1050094223,(Frog_T2R10--Intact_1/1-284:0.0295313122,Frog_T2R32--Intact_1/1-
284:0.0758918865)0.9998500075:0.1040340316)0.9998500075:0.2634336645,(Frog_T2R39--Intact_1/1-
285:0.1451722830,(Frog_T2R37--Intact_1/1-287:0.1313889101,Frog_T2R41--Intact_1/1-
287:0.1546973339)0.9908442764:0.0365337228)0.9998500075:0.2693605432)0.9524055806:0.070190
8914)0.9998500075:0.7247662569,((Frog_T2R43--Intact_1/1-279:0.0276156267,(Frog_T2R44--
Intact_1/1-279:0.0762978677,Frog_T2R45--Intact_1/1-
283:0.1242370242)0.9998500075:0.0807449944)0.9998500075:0.6281820276,((Frog_T2R20--
Intact_1/1-287:0.3367721774,(Frog_T2R17--Intact_1/1-267:0.1836411932,(Frog_T2R13--Intact_1/1-
284:0.1429331211,Frog_T2R15--Intact_1/1-
286:0.1908147297)0.9988383537:0.0622540761)0.9998500075:0.2489369796)0.9998500075:0.580226
5024,((Frog_T2R14--Intact_1/1-279:0.2509771371,(Frog_T2R18--Intact_1/21-
297:0.0494714498,Frog_T2R19--Intact_1/1-
260:0.0303239435)0.9998500075:0.1444025520)0.9998500075:0.2125366467,(Frog_T2R16--Intact_1/1-
267:0.4952301394,Frog_T2R21--Intact_1/1-
274:0.6604838105)0.9776221557:0.1364395644)0.9998500075:0.2881570838)0.9998500075:0.216352
0704)0.7776615141:0.0984982343)0.4476848763:0.0280234836,(((Fugu_T2R2--Intact_1/1-
281:0.6132557313,(Zebrafish_T2R1--Intact_1/1-293:0.0574349317,Zebrafish_T2R2--Intact_1/11-
304:0.1261576988)0.9998500075:0.3243151406)0.9998500075:0.9257419205,
((LC_T2R01_g03562_fishlike/1-292:0.4385990364,(Stickleback_T2R3--Intact_1/1-303:0.0791503986,

(Fugu_T2R1--Intact_1/1-302:0.0869870169,Pufferfish_T2R1--Intact_1/1-
302:0.1825167872)0.9998500075:0.1475367072)0.9998500075:0.5454529512)0.9998500075:0.747070
1070,(Zebrafish_T2R3--Intact_1/1-278:0.6597843260,(Zebrafish_T2R4--Intact_1/3-272:0.9018007676,
((Fugu_T2R4--Intact_1/1-263:0.3698918948,Pufferfish_T2R6--Intact_1/1-
260:0.2518950626)0.9998500075:0.5707499799,((Stickleback_T2R1--Intact_1/3-
272:0.0735748762,Stickleback_T2R2--Intact_1/3-268:0.0278038677)0.9998500075:0.4303115318,
(Fugu_T2R3--Intact_1/3-269:0.2505449509,(Pufferfish_T2R3--Intact_1/3-268:0.1928170449,
(Pufferfish_T2R4--Intact_1/1-266:0.0817819622,(Pufferfish_T2R2--Intact_1/1-
269:0.0244307538,Pufferfish_T2R5--Intact_1/1-
269:0.0254920824)0.9998500075:0.1391987942)0.9998500075:0.0652164303)0.9998500075:0.153993
9690)0.9998500075:0.2126456684)0.9998500075:0.2688511494)0.9669210246:0.0978596136)0.97672
03407:0.1183212442)0.9998500075:0.8027202107)0.6524222508:0.0700574430)0.9256339886:0.12242
84761,((LC_T2R02_JH128442-389500-390500/1-287:0.5919641727,
(LC_T2R03_ENSLACG00000000459/5-289:0.3529263945,LC_T2R04_ENSLACG00000005021/1-
292:0.2907779392)0.9998500075:0.4349795895)0.9998500075:0.1843612242,
(LC_T2R05_ENSLACG00000002015/1-302:0.3297067428,(LC_T2R06_ENSLACG00000000717/1-
297:0.1804097940,((LC_T2R07_ENSLACG00000000429/1-295:0.0864375690,(LC_T2R08-r_JH130784-
23237-24160/1-299:0.1292119929,(LC_T2R09_ENSLACG00000003709/9-307:0.0632866935,
(LC_T2R10_ENSLACG00000002588/1-300:0.0451538179,(LC_T2R11_ENSLACG00000000516/1-
296:0.0183791268,LC_T2R12_JH130784-b58450-b59400/4-
303:0.0470670107)0.9998500075:0.0109042506)0.9998500075:0.0397194455)0.9991873003:0.036271
4749)0.9358963985:0.0267032814)0.9998500075:0.3365818215,((LC_T2R13_JH130976-3613-4524/1-
300:0.3070308455,((LC_T2R14_g02292/13-312:0.0289168699,LC_T2R15_JH129824-107559-108476/1-
300:0.0186604016)0.9998500075:0.0416612994,((LC_T2R16_g02330/13-
312:0.0257659952,LC_T2R17_g02899/1-300:0.0222969243)0.9998500075:0.0245928735,
(LC_T2R18_JH131589-29724-30617/9-294:0.0675650722,LC_T2R19-124199-125143/1-
300:0.0154652652)0.9998500075:0.0281562755)0.3032687553:0.0163665249)0.9998500075:0.364698
5846)0.9909907968:0.0370578107,((LC_T2R20_g00491/11-310:0.0469882582,LC_T2R21_g05130/13-
304:0.0030171745)0.9998500075:0.1627111116,((LC_T2R22_JH129648-127088-127993/1-
300:0.1747709926,LC_T2R23_ENSLACG00000001021/1-
290:0.2764590245)0.9998500075:0.0713104869,(LC_T2R24_ENSLACG00000001744/4-
304:0.2534522961,((LC_T2R25_JH129648-116200-117050/1-297:0.1600651602,
(LC_T2R26_JH130917-46105-46999/1-295:0.1042439784,(LC_T2R27_ENSLACG00000001874/1-
300:0.1630784674,((LC_T2R28_g06895/1-299:0.0878065142,Lc_T2R29_JH128085-30766-31662/1-
295:0.0765555986)0.9998500075:0.1286341710,(LC_T2R30_ENSLACG00000001246/1-
299:0.0723310449,(LC_T2R31_JH128085-329000-329900/1-300:0.0481077861,LC_T2R32_JH128085-
412667-413584/1-

299:0.0930470872)0.9449286383:0.0148181688)0.9998500075:0.1209767063)0.9998500075:0.046450
0873)0.9998500075:0.0394929695)0.9998500075:0.0931800935)0.9998500075:0.0469522097,
(((LC_T2R33_g01751/1-294:0.1580192460,((LC_T2R34_JH129988-117000-119000/1-
295:0.0790185626,LC_T2R35_ENSLACG00000000678/13-
302:0.0684089244)0.9998500075:0.0741050618,(LC_T2R36_g01331/1-
297:0.0645545122,LC_T2R37_JH129988-167404-168291/1-
295:0.0721536692)0.9998500075:0.0673071302)0.9876579378:0.0330008929)0.9998500075:0.216258
6537,(LC_T2R38_ENSLACG00000000317/13-303:0.1822088658,(LC_T2R39_g01372/11-
301:0.2046444232,LC_T2R40_ENSLACG00000002445/1-
301:0.2124071906)0.9988822015:0.0406950753)0.9998500075:0.1128563153)0.9998500075:0.0961676
211,((LC_T2R41_JH130077-122561-123463/1-
297:0.0424660428,LC_T2R42_ENSLACG00000006135/4-
293:0.0143747203)0.9998500075:0.1921306702,((LC_T2R43_JH132281-3349-42091/1-
283:0.0413513239,LC_T2R44_ENSLACG00000005213/2-
296:0.0161512404)0.9998500075:0.2213721895,((((LC_T2R45_ENSLACG00000002953/2-
296:0.1895152970,(LC_T2R46_JH132617-3356-4267/1-299:0.1004729088,(LC_T2R47_JH134280-
2000-3700/1-299:0.0185803549,LC_T2R48_JH131192-13968-14870/1-
296:0.0872585676)0.9998500075:0.0367990437)0.9998500075:0.2113378609)0.9998500075:0.0591262
848,(LC_T2R49_g03496/1-300:0.3438861481,(LC_T2R50_JH135454-2600-3600/1-
295:0.1511502456,LC_T2R51_JH130976-56756-57640/1-
292:0.0490532745)0.9998500075:0.0885755659)0.9938065021:0.0463101885)0.9998500075:0.093454
5582,((LC_T2R52_ENSLACG00000002071/2-301:0.0229632341,LC_T2R53_ENSLACG00000002046/1-
293:0.0013143107)0.9998500075:0.1124191901,((LC_T2R54_JH130641-11547-12443/1-
295:0.0109428000,Lc_T2R55_JH130827-7169-8065/1-293:0.0638314445)0.9998500075:0.0572051262,
(LC_T2R56_ENSLACG00000001061/14-304:0.1897064198,LC_T2R57-AFYH01284138-450-1400/1-
295:0.1031690167)0.9998500075:0.0347557749)0.9998500075:0.1239290370)0.7342368583:0.023392
2149)0.9998500075:0.0761066055,(LC_T2R58_g06318/2-294:0.1965316438,(LC_T2R59_JH128916-
126493-127395/1-295:0.1961340514,((LC_T2R60_JH128916-233505-234389/1-293:0.1763913703,
(Lc_T2R61_JH128916-195708-196628/1-299:0.0554555418,Lc_T2R62_JH129837-106889-107800/1-
296:0.0452095784)0.9998500075:0.1081398499)0.9998500075:0.0575370598,
((LC_T2R63_ENSLACG00000002255/3-302:0.2685466036,LC_T2R64_ENSLACG00000003395/1-
295:0.1401749008)0.9998500075:0.1802666677,((LC_T2R65_JH131022_JH1310226900-7950/1-
297:0.0891894585,(LC_T2R66_JH128085-553378-554280/1-
297:0.0857935224,LC_T2R67_ENSLACG00000002847/21-
319:0.0970832888)0.7413427987:0.0105894874)0.9998500075:0.0979740923,(((LC_T2R68_JH129750-
41217-42131/1-300:0.0144234751,LC_T2R69_ENSLACG00000003809/1-
300:0.0343516777)0.9998500075:0.0642377312,((LC_T2R70_JH129303-210000-211000/1-

297:0.0957336846,LC_T2R71_ENSLACG00000003104/2-
301:0.1053856875)0.9998500075:0.0672593022,((LC_T2R72_JH129303-64562-65465/1-
296:0.1009076620,(LC_T2R73_JH129303-155997-156905/4-300:0.0559599606,LC_T2R74_g01418/1-
294:0.0552175654)0.9965810479:0.0383443495)0.9914567671:0.0410884218,(LC_T2R75_JH129303-
138585-139493/2-299:0.0638237132,LC_T2R76_ENSLACG00000007023/1308/1-
300:0.0569957096)0.9998500075:0.0388665336)0.8968498631:0.0222643589)0.9998500075:0.034998
8040)0.9998500075:0.0558306118,((LC_T2R77_g03692/2-294:0.0472445084,LC_T2R78_g07306/2-
291:0.0583707446)0.9998500075:0.1083160654,(LC_T2R79_ENSLACG00000003761/4-
299:0.1032237258,LC_T2R80_JH128916-42068-43018/1-
298:0.2479923501)0.9986466810:0.0331618003)0.9998500075:0.0387142154)0.7134418367:0.014864
8121)0.9998500075:0.0334328316)0.9196285222:0.0230334188)0.8889689453:0.0066205372)0.99985
00075:0.0370058722)0.9998500075:0.0409882192)0.9950076293:0.0258306619)0.9998500075:0.0655
325789)0.9998500075:0.1844516044)0.9985305260:0.0389475213)0.9998500075:0.0425943265)0.516
8534148:0.0221038740)0.9998500075:0.0365085037)0.9998500075:0.0942397554)0.9915365280:0.05
28703830)0.9946937185:0.0448536600)0.9199640692:0.0733186720)0.9998500075:0.3933329995)0.9
938695461:0.1452443399)0.9998500075:0.2925871036)0.9986107237:0.0947908125)0.9998500075:0.
1484348876)0.9998500075:0.9264217956)0.8325675189:0.0634882284)0.9998500075:0.3703017821);

Part 5 Tree file in Newick format for T2R and V1R sequences shown in Fig. 3.

(Tn_ORA6/1-274:0.0723508757,Tr_ORA6/1-274:0.0556883993,(Ga_ORA6/1-279:0.2991686178,(Ol_ORA6/1-267:0.3311611620,(Dr_ORA6/1-271:0.4657094175,(LC-V1R06-JH127233-161522-162418/1-271:0.6036148467,((Dr_ORA5/1-272:0.3090329600,(Ol_ORA5/1-276:0.2424578650,(Ga_ORA5/1-276:0.1202849751,(Tn_ORA5/1-276:0.0438863580,Tr_ORA5/1-276:0.0784272507)0.9998500075:0.0744012656)0.6131671097:0.0376778491)0.9998500075:0.1869032519)0.9998500075:0.6464476045,(LC-V1R05-JH132282-2162-3064/1-288:1.0976355200,(((Pm_Seq2/1-291:0.4508625953,((Dr_ORA4/1-302:0.1271259818,(Ga_ORA4/1-304:0.1159473055,(Ol_ORA4/1-306:0.1171404927,(Tn_ORA4/1-303:0.0425557892,Tr_ORA4/1-306:0.0271570701)0.9998500075:0.0639837369)0.9986198423:0.0274309327)0.9333401636:0.0505262868)0.9998500075:0.4826613930,(LC-V1R04-JH126562-9351125-9352069/1-291:0.5155202558,((Dr_ORA3/14-309:0.2074971297,(Ol_ORA3/22-317:0.1329113576,(Ga_ORA3/38-331:0.1013542757,(Tn_ORA3/18-312:0.0510650474,Tr_ORA3/20-315:0.0384099615)0.9998500075:0.0620046823)0.9998500075:0.0960520345)0.9956555058:0.0810097271)0.9998500075:0.3408357989,(LC-V1R03-JH126562-9337639-9338598/1-296:0.2699466062,Xt_ORA15/1-290:0.5285490068)0.9998500075:0.1168869700)0.9935878895:0.0811746587)0.7622181333:0.0616582515)0.9985080143:0.1071481456)0.9998500075:0.3714679276,(Pm_Seq3/29-298:1.0701172738,Pm_Seq4/4-277:0.9234218796)0.6543355936:0.1279256372)0.9300504799:0.1288375071,(Pm_Seq1/1-295:1.0099648534,((LC-V1R02-g18669/2-307:0.2557173550,(Dr_ORA2/1-299:0.3560633098,(Ol_ORA2:0.2402654527,(Ga_ORA2/1-303:0.1425624429,(Tn_ORA2:0.0959926370,Tr_ORA2:0.0472976064)0.9998500075:0.1108984838)0.8856725377:0.0341203966)0.9481716559:0.0597035292)0.9998500075:0.3685646708)0.9577202560:0.0874505557,(LC-V1R07-g18654/1-305:0.3060566256,(((LC-V1R08-JH126576-3899564-3900517/1-304:0.4328010479,(LC-V1R09-JH126576-3966530-3967480/1-304:0.0987021363,LC-V1R10_JH126576-3956453-3957412/1-305:0.0923615432)0.9998500075:0.1444836694)0.9919266682:0.0499868587,(Xt_ORA12/1-305:0.3946953274,(Xt_ORA13/1-305:0.3887812397,((Xt_ORA10/1-305:0.0935138433,(Xt_ORA9/1-309:0.0703050012,Xt_ORA8/2-310:0.0556103021)0.9998500075:0.0381433721)0.9998500075:0.1320874924,((Xt_ORA6/16-320:0.2113119306,(Xt_ORA2/1-305:0.0005341247,(Xt_ORA5/100-404:0.0387133920,(Xt_ORA3/1-282:0.0093730281,Xt_ORA4/1-305:0.0467720908)0.9998500075:0.0168804645)0.9998500075:0.0355076735)0.9998500075:0.156686

6611)0.9998500075:0.0831198796,(Xt_ORA11/1-203:0.5006639947,Xt_ORA7/1-
302:0.1724031260)0.8300370250:0.0344629407)0.9998500075:0.0484705497)0.8881500064:0.070187
4234)0.9998500075:0.1369920133)0.9998500075:0.1401683079)0.9998500075:0.1772626764,((LC-
V1R11-g13453/2-306:0.5124178961,(LC-V1R12-JH127167-62652-63599/1-305:0.3475570248,((LC-
V1R13-g01824/1-305:0.2116315934,LC-V1R14-g02196/1-
305:0.1415482785)0.9868942290:0.0294676668,((LC-V1R15-JH132386-1786-2700/1-
296:0.0141683996,LC-V1R16-g00870/2-305:0.0356775263)0.9998500075:0.2167276768,(LC-V1R17-
g00958/2-304:0.0604363990,(LC-V1R18-g00627/2-304:0.0243492288,LC-V1R19-JH129249-102462-
103379/1-
298:0.1573391493)0.9998500075:0.0976766452)0.9998500075:0.1895622681)0.9998500075:0.229495
6009)0.9998500075:0.0608931502)0.9998500075:0.0916700631)0.9998500075:0.1273264606,((LC-
V1R01-JH126576-3892974-3893936/1-303:0.3466896907,(LC-V1R01a-g18646/2-306:0.1302384955,
(Xt_ORA1/1-302:0.3330458332,(Dr_ORA1:0.1456929731,(Ol_ORA1/1-302:0.0617918733,Ga_ORA1/1-
301:0.0714317881)0.9998500075:0.0730489916)0.9998500075:0.1188779578)0.9846869500:0.0523549
069)0.9917444362:0.0515043193)0.9289253977:0.0608016460,(Xt_ORA14/1-297:0.7007858137,
(((Vmn1r87/1-285:0.4792637275,(Vmn1r85/2-300:0.2912781274,(Vmn1r89/1-306:0.0723560874,
(Vmn1r86/1-306:0.0259951370,Vmn1r88/1-
306:0.1031998438)0.9961572544:0.0256583832)0.9998500075:0.2177615533)0.9998500075:0.155292
6322)0.9998500075:0.4150819770,(((Vmn1r195/1-305:0.2517977719,((Vmn1r216/3-297:0.0811790786,
(Vmn1r193/20-318:0.0519904971,Vmn1r204/20-
318:0.0691802920)0.9998500075:0.0366129581)0.9998500075:0.1329225258,(Vmn1r194/1-
295:0.1937216777,Vmn1r215/1-
299:0.1659140752)0.9998500075:0.0703225991)0.8199219115:0.0207231632)0.9998500075:0.2139339
569,(Vmn1r211/1-297:0.2418752419,(Vmn1r196/1-299:0.2676358971,(Vmn1r200/1-301:0.0792464552,
((Vmn1r206/1-301:0.0367725961,Vmn1r207/1-301:0.0390411406)0.9998500075:0.0568049374,
(Vmn1r208/1-295:0.1288720127,(Vmn1r199/44-346:0.1085449077,(Vmn1r212/1-
302:0.1105056615,Vmn1r222/1-
301:0.1157273483)0.9998500075:0.0491708133)0.9948234594:0.0260720078)0.9861159689:0.0152082
945)0.9147624691:0.0112252913)0.9998500075:0.1194970073)0.9998500075:0.0909009910)0.9998500
075:0.2338975167)0.9998500075:0.3331540459,(((Vmn1r19/1-299:0.0790056475,(Vmn1r18/1-
299:0.0499008348,Vmn1r28/1-
299:0.0387974599)0.9998500075:0.0358132081)0.9880884805:0.0421577602,((Vmn1r25/1-
299:0.1548342214,(Vmn1r22/1-299:0.0400527618,Vmn1r23/1-
299:0.0384558861)0.9998500075:0.0787736327)0.9998500075:0.0345308843,(((Vmn1r11/1-
299:0.0606066753,((Vmn1r32/1-299:0.0237307874,(Vmn1r33/1-299:0.1072977457,(Vmn1r35/1-
296:0.0229010398,Vmn1r36/1-
299:0.0567386161)0.9998500075:0.0465449283)0.9945709836:0.0160712605)0.9998500075:0.050872

4234,(Vmn1r24/1-296:0.0988134537,Vmn1r26/1-
291:0.1025322905)0.9998500075:0.0402635237)0.2317190862:0.0109891270)0.9960947766:0.008658
1264,(Vmn1r4/1-297:0.1150980581,((Vmn1r5/1-299:0.0566198828,Vmn1r6/1-
299:0.0405398331)0.9998500075:0.0568345666,((Vmn1r16/1-299:0.0525017181,Vmn1r29/1-
299:0.0529851716)0.9998500075:0.0445645461,(((Vmn1r9/1-299:0.0100534087,Vmn1r10/1-
299:0.0093490375)0.9998500075:0.0512528305,(Vmn1r7/1-299:0.0271144005,Vmn1r8/1-
299:0.0096057010)0.9998500075:0.0582059312)0.9998500075:0.0498709355,((Vmn1r20/1-
299:0.0108645650,Vmn1r27/1-299:0.0182640033)0.9998500075:0.0754952192,(Vmn1r17/1-
299:0.0317444804,Vmn1r31/1-
299:0.0564153533)0.9998500075:0.0294385974)0.9998500075:0.0330072814)0.9985512137:0.018340
0574)0.9878142440:0.0095688850)0.9352454011:0.0087691266)0.9998500075:0.0179067680)0.99985
00075:0.0173535768,((Vmn1r12/1-301:0.0593199466,Vmn1r14/1-
297:0.0425493498)0.9998500075:0.0723479111,(Vmn1r13/1-299:0.1071054158,Vmn1r15/1-
299:0.1164837751)0.9991786807:0.0205151049)0.9998500075:0.0123469683)0.9601134311:0.0095485
499)0.9921362255:0.0615925844)0.9998500075:0.3625908712,(Vmn1r1/1-300:0.3678383857,
((Vmn1r44/1-302:0.0525487535,((Vmn1r50/1-302:0.1146460281,Vmn1r49/1-
302:0.0695098154)0.9851658824:0.0220138687,(Vmn1r40/1-302:0.0631465839,Vmn1r41/2-
303:0.0864742592)0.9998500075:0.0227639927)0.9372309752:0.0220290454)0.9998500075:0.229913
7128,(Vmn1r54/17-317:0.3399697648,((Vmn1r47/1-302:0.0327138501,Vmn1r48/1-
296:0.0104848306)0.9998500075:0.0422733652,(Vmn1r42/17-318:0.1161568022,Vmn1r45/17-
312:0.0380879633)0.9930590098:0.0256488191)0.9998500075:0.1776416872)0.5260260219:0.027553
9806)0.9998500075:0.1415590550)0.9998500075:0.2543560654)0.9998500075:0.3132721980)0.91755
07332:0.0871302627)0.1956379228:0.1105816811,(((((Vmn1r77/3-305:0.1881036678,(Vmn1r79/3-
305:0.0000000001,(Vmn1r2/3-305:0.0140531152,Vmn1r3/3-
305:0.0084405522)0.9998500075:0.0056029435)0.9998500075:0.1184102100)0.9998500075:0.0409000
311,((Vmn1r78/3-307:0.1668450870,(Vmn1r81/3-305:0.0986548090,Vmn1r83/3-
305:0.0709572635)0.9998500075:0.0487574356)0.9998500075:0.0351266429,(Vmn1r75/3-
304:0.1376997517,(Vmn1r73/1-302:0.1711654717,(Vmn1r76/21-323:0.0962515896,(Vmn1r74/1-
303:0.1141712771,Vmn1r80/1-
305:0.0682440751)0.7861408523:0.0094040036)0.9998500075:0.0537217150)0.8471209809:0.007096
3456)0.7304198548:0.0173103077)0.9887959095:0.0267687629)0.9998500075:0.1739167740,
(Vmn1r82/1-303:0.2574597774,Vmn1r84/13-
317:0.3430922283)0.3102928016:0.0385826977)0.9998500075:0.2811986390,((((Vmn1r66/6-
309:0.1972142934,(Vmn1r67/1-304:0.1667911127,(Vmn1r68/15-318:0.0680505990,Vmn1r69/15-
318:0.0506099718)0.9998500075:0.1138920245)0.9607275087:0.0234218963)0.9998500075:0.0462261
074,(Vmn1r71/1-305:0.2180246372,(Vmn1r184/5-309:0.0514135789,Vmn1r185/6-
310:0.0427106981)0.9998500075:0.1802289652)0.9998500075:0.0669155563)0.9998500075:0.129151

7467,(Vmn1r224/1-297:0.3284587327,(Vmn1r227/1-304:0.2152222881,(Vmn1r228/31-
332:0.1441618617,Vmn1r232/46-
349:0.1284858568)0.9647599877:0.0408094026)0.9998500075:0.1954317056)0.8426622901:0.042581
4022)0.9998500075:0.1719303244,(Vmn1r234/24-328:0.4195878651,Vmn1r237/1-
289:0.2661119680)0.9998500075:0.1220304071)0.9998500075:0.1892207076)0.9803453771:0.1061173
015,(Vmn1r172/1-309:0.2287917479,(((Vmn1r56/1-295:0.0467831068,(Vmn1r57/1-295:0.0355666238,
(Vmn1r59/1-295:0.0413967514,(Vmn1r58/1-295:0.0275662636,((Vmn1r62/1-
295:0.0122275190,Vmn1r63/1-295:0.0225556232)0.9839700885:0.0035333665,(Vmn1r60/1-
295:0.0171962706,Vmn1r61/1-
295:0.0538633806)0.9998500075:0.0199104368)0.9998500075:0.0152811450)0.9998500075:0.0459860
270)0.8839316864:0.0094650521)0.9998500075:0.0255830208)0.9998500075:0.2199284453,
(Vmn1r171/13-314:0.0077254560,(Vmn1r176/1-302:0.0706148933,Vmn1r175/1-
302:0.0256745083)0.6216842301:0.0208500366)0.9998500075:0.1609578333)0.9659322286:0.032743
7113,((Vmn1r90/1-307:0.0412264411,(Vmn1r168/1-307:0.0155403664,Vmn1r177/1-
307:0.0125459585)0.9998500075:0.0363331391)0.9998500075:0.1896569114,(Vmn1r178/1-
302:0.0974402593,((Vmn1r181/1-302:0.0549479436,Vmn1r182/1-
303:0.0903009690)0.9998500075:0.0479570889,((Vmn1r102/1-309:0.0093248067,(Vmn1r93/1-
309:0.0000000001,Vmn1r126/1-
309:0.0109478971)0.9998500075:0.0072279871)0.9998500075:0.0689069125,(((Vmn1r105/1-
305:0.0185366955,(Vmn1r129/1-305:0.0157220819,Vmn1r96/1-
305:0.0150278585)0.5568981164:0.0048077150)0.9998500075:0.0193798021,((Vmn1r98/1-
305:0.0055507653,Vmn1r119/1-305:0.0055424552)0.9937597198:0.0030830843,(Vmn1r92/1-
305:0.0085415045,Vmn1r101/1-
305:0.0250624052)0.9952198521:0.0083284881)0.9998500075:0.0109263586)0.9998500075:0.049163
6294,(((Vmn1r103/1-303:0.0170667543,(Vmn1r99/1-303:0.0028814685,Vmn1r120/1-
303:0.0198239279)0.9998500075:0.0228552805)0.9998500075:0.0086413154,(Vmn1r94/1-
303:0.0112905387,Vmn1r127/1-
303:0.0084037948)0.9654221063:0.0028062959)0.9998500075:0.0235412086,(Vmn1r123/1-
305:0.0363101918,((Vmn1r106/1-304:0.0261015879,(Vmn1r97/1-304:0.0035404657,Vmn1r108/1-
304:0.0076517974)0.9998500075:0.0246183523)0.9998500075:0.0216867184,((Vmn1r91/1-
305:0.0171485351,Vmn1r124/1-305:0.0309646224)0.9998500075:0.0112004427,(Vmn1r109/1-
295:0.0204479264,(Vmn1r100/1-305:0.0033203214,(Vmn1r125/1-305:0.0088819303,(Vmn1r128/1-
305:0.0167836975,(Vmn1r95/1-305:0.0027769195,Vmn1r104/1-
305:0.0111202794)0.9822272720:0.0027594872)0.9998500075:0.0162638696)0.9988490838:0.0055610
804)0.9998500075:0.0050232555)0.9737422723:0.0030847422)0.9998500075:0.0185144111)0.9998500
075:0.0104042281)0.9806874196:0.0092107280)0.9998500075:0.0158432618)0.9998500075:0.0243311
922)0.9998500075:0.0265631805)0.7313167105:0.0144399375)0.9998500075:0.1713000963)0.963435

2319:0.0398731916)0.9948001940:0.0718521388)0.9998500075:0.7961031891)0.9998500075:0.16686
13512)0.9998500075:0.2932332395)0.9998500075:0.2100817075)0.9998500075:0.1337883240)0.9998
500075:0.0657077723)0.9998500075:0.0642403945)0.9998500075:0.1036045789)0.9998500075:0.495
9055916)0.9893960458:0.1660206901)0.9938415140:0.2053234115)0.9952980337:0.2476082493)0.99
98500075:0.5838714662)0.9998500075:0.3643861020)0.9980247704:0.1718649571)0.6620329349:0.0
661651291)0.9998500075:0.1747632360);
